# Nonsense Mutations in the Yeast *SUP35* Gene Affect the [*PSI^+^*] Prion Propagation

**DOI:** 10.3390/ijms21051648

**Published:** 2020-02-28

**Authors:** Nina P. Trubitsina, Olga M. Zemlyanko, Stanislav A. Bondarev, Galina A. Zhouravleva

**Affiliations:** 1 Department of Genetics and Biotechnology, Saint Petersburg State University, 199034 St. Petersburg, Russia; n.trubitsina@spbu.ru (N.P.T.); o.zemlyanko@spbu.ru (O.M.Z.); s.bondarev@spbu.ru (S.A.B.); 2 Laboratory of Amyloid Biology, Saint Petersburg State University, 199034 St. Petersburg, Russia

**Keywords:** yeast, *S.cerevisiae*, prion, [*PSI*^+^], translation termination, Sup35, nonsense mutations

## Abstract

The essential *SUP35* gene encodes yeast translation termination factor eRF3. Previously, we isolated nonsense mutations *sup35-n* and proposed that the viability of such mutants can be explained by readthrough of the premature stop codon. Such mutations, as well as the prion [*PSI*^+^], can appear in natural yeast populations, and their combinations may have different effects on the cells. Here, we analyze the effects of the compatibility of *sup35-n* mutations with the [*PSI*^+^] prion in haploid and diploid cells. We demonstrated that *sup35-n* mutations are incompatible with the [*PSI^+^*] prion, leading to lethality of *sup35-n* [*PSI^+^*] haploid cells. In diploid cells the compatibility of [*PSI^+^*] with *sup35-n* depends on how the corresponding diploid was obtained. Nonsense mutations *sup35-21*, *sup35-74,* and *sup35-218* are compatible with the [*PSI^+^*] prion in diploid strains, but affect [*PSI^+^*] properties and lead to the formation of new prion variant. The only mutation that could replace the *SUP35* wild-type allele in both haploid and diploid [*PSI^+^*] strains, *sup35-240*, led to the prion loss. Possibly, short Sup35_1–55_ protein, produced from the *sup35-240* allele, is included in Sup35 aggregates and destabilize them. Alternatively, single molecules of Sup35_1–55_ can stick to aggregate ends, and thus interrupt the fibril growth. Thus, we can conclude that *sup35-240* mutation prevents [*PSI^+^*] propagation and can be considered as a new *pnm* mutation.

## 1. Introduction

*SUP35* gene in *Saccharomyces cerevisiae* yeast encodes translation termination factor eRF3, and thus it is an essential gene. During translation termination eRF3 interacts with a product of another essential gene, *SUP45*, which encodes a translation termination factor eRF1. The essential nature of both genes implies that nonsense mutations in each of them should be lethal. However, previously we isolated such mutations, designated them as *sup35-n* or *sup45-n* (*n* – from nonsense) and proposed that the viability of such mutants can be explained by readthrough of the premature stop codon (PTC) because of the decreased amount of one translation termination factor [1,2]. In support of this assumption, we showed that all *sup35-n* and *sup45-n* mutants contain a decreased amount of full-length eRF3 or eRF1, respectively, together with short fragments synthesized in the case of translation termination on PTC [1,2]. Similar mutations have also been isolated in other laboratories (reviewed in [3]). The amount of full-length eRF3 in *sup35-n* mutants was very low, for example *sup35-240* and *sup35-218* mutants contained 6% and 8% of full-length eRF3, respectively, compared to the wild-type cells [2]. This was also the case for full-length eRF1 in the *sup45-n* mutants [1]. However, a more than ten-fold decrease in eRF3 or eRF1 did not affect the viability of the cells significantly, suggesting that a very low level of either translation termination factor (eRF1 or eRF3) is sufficient for cell viability [2]. However, it remains unclear how yeast cells can survive in the absence of the normal quantities of essential protein. Interestingly, that depletion of eRF3 in *sup35-n* mutants was not accompanied by a reduction in the eRF1 level, and vice versa—nonsense mutations in *SUP45* did not lead to reduced levels of eRF3 [2]. Such observations are unexpected, as previously it was shown that repression of *SUP35C* or *SUP45* genes expression resulted in decreases in the levels of both eRF1 and eRF3, accompanied by reductions in yeast cell viability [4]. The disparity between spontaneous nonsense mutants and an artificially constructed system may be caused by differences in the approaches that have been used [2].

The matter becomes more complicated if we take into account the fact that Sup35 is able to form a self-perpetuating amyloid-like aggregates, giving rise to the [*PSI^+^*] prion. Yeast Sup35 consists of three domains (N, M, and C) whose boundaries were assigned to the second and the third methionine residues, respectively. The N- and M-domains of eRF3 are not essential for viability and translation termination, in contrast to the essential C-proximal region [5]. The N-domain of Sup35 (Sup35N) is responsible for generation and propagation of the [*PSI^+^*] prion. Aggregation of Sup35 in the [*PSI^+^*] cells results in defective translation termination that leads to omnipotent nonsense suppression (reviewed in [6]). [*PSI^+^*] prion exists in different variants (“strains”) [7,8].

Previously we have shown that the combination of strong [*PSI*^+^] with *sup45-n* mutations leads to the synthetic lethality that was detected even in the heterozygous state. This lethal effect was explained by a too high readthrough level in the cells combining a decreased amount of Sup45 with a decreased amount of functional Sup35 [9].

In current study we have analyzed the interplay between two nonsense suppressors: *sup35* nonsense mutations and the [*PSI*^+^] factor. This problem is very complex because of several reasons. First, both factors have common phenotypic manifestation. Second, low level of the Sup35 protein caused by *sup35-n* can affect cell viability itself and can be modified by the prion. Third, truncated fragments of Sup35 are present in *sup35-n* cells together with the full-length Sup35 protein. Despite all these difficulties, we tried to investigate this complex issue, since it may be relevant for the natural yeast populations. The sequence of *SUP35* (especially its part encoding the N-domain of the protein) is enriched by potential nonsense mutation sites (reviewed in [3]) that can lead to increased frequencies of *sup35-n* mutations. Indeed, among 48 spontaneous *sup35* mutations one-third was characterized by a decrease in the amount of Sup35 [2]. Based on these data, it can be assumed that *sup35-n* mutations can often be present in natural yeast populations. The [*PSI*^+^] prion was also found in natural populations of yeasts [10]. Thus, in wild yeast strains the prion can be easily combined with *sup35-n* by chance, but the fate of such cells is unknown.

Here, we demonstrate that, unlike *sup45-n*, *sup35* nonsense mutations do not lead to synthetic lethality (or incompatibility) when plasmid with wild-type *SUP35* gene is present. However, loss of this plasmid, leads to lethality of *sup35-n* [*PSI^+^*] haploid cells. In diploid cells the compatibility of [*PSI^+^*] with *sup35-n* depends on how the corresponding diploid was obtained, suggesting that during the cultivation of [*psi^−^*] *sup35-n* haploids, additional mutations may be selected. We also describe a new *pnm* mutation that induces [*PSI^+^*] loss in both haploid and diploid cells.

## 2. Results

### 2.1. Characterization of Nonsense Mutations sup35-n

**Figure 1:**
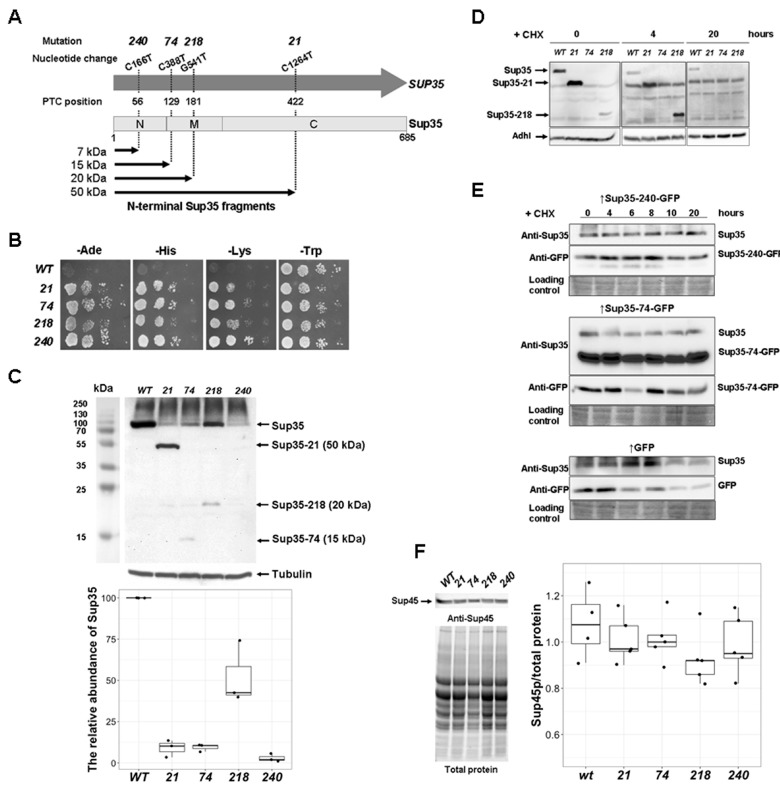
Nonsense mutations in the *SUP35* gene lead to an omnipotent nonsense suppression and a decrease in the amount of full-length Sup35. (**A**) Localization of nonsense mutations in *SUP35*. PTC—premature termination codon. N, M, and C—Sup35 domains. (**B**) All *sup35-n* mutations lead to strong omnipotent suppression. Growth of haploid [*psi^−^*] cells with different *sup35-n* mutations on media for the detection of nonsense suppression after 5 days of incubation at 30 °C. Growth on media without tryptophan (-Trp) was used as a control. Ten-fold serial dilutions are shown. Numbers on the left correspond to *sup35-n* mutations. (**C**) Lysates of the strains shown on the Panel B were analyzed by Western blotting with anti-Sup35 and anti-Tubulin antibodies. The numbers above the lanes indicate the *sup35* mutation. kDa—protein molecular weight marker. Fragments corresponding to the full-length and truncated Sup35 are marked by the arrows. Tubulin was used as a loading control. The plot below shows the amount of Sup35 in *sup35-n* mutants relative to the wild type strain. Dots correspond to the independent replicates. Amount of Sup35 in the wild-type (*WT*) is taken as 100%. (**D**) Steady-state level of Sup35 in yeast cells containing wild-type copy of *SUP35* (*WT*) or nonsense mutations. Sup35 was estimated in 7A-D832 strain bearing pRSU1 plasmid with either *WT SUP35* or one of the mutant *sup35-n* alleles after incubation in YPD medium containing 100 µg mL^−1^ cycloheximide (CHX) during 0, 4, or 20 h. The same strains as on the Panel B were used, in the case of *sup35-74* truncated Sup35 fragment was not detected. Adh1 was used as a loading control. (**E**) Steady-state level of Sup35-240-GFP and Sup35-74-GFP in yeast cells containing wild-type copy of *SUP35*. For the *CUP1* promoter induction cells were grown in selective medium containing 50 µM CuSO_4_ during 2 h. The amount of Sup35 or its fragments fused with GFP was estimated in 7A-D832 strain bearing pRSU1 plasmid with wild-type *SUP35* in combination of pRS316-pCUP-SUP35-240-GFP (Sup35-240-GFP) or pRS316-pCUP-SUP35-74-GFP (Sup35-74-GFP) after incubation in SC-Leu-Ura medium containing 100 µg mL^−1^ cycloheximide (CHX) during 0 ‒ 20 h. pRS316-pCUP-GFP (GFP) was used as a control. Coomassie staining of the same gel was used as a loading control. (**F**) Strains with *sup35-n* mutations have the same Sup45 level. Sup45 quantity was normalized to the total protein in the same gels using gel documentation system (ChemiDoc XRS+ System, Bio-Rad, Hercules, CA, USA). The difference between amount of Sup45 for WT and *sup35-n* mutants in Wilcoxon rank-sum test was not found. Dots correspond to the independent replicates.

In this study we selected four mutant alleles of the *SUP35* gene among *sup35-n* mutations described earlier [2]. The main criterion for selection was their localization in different parts of *SUP35*, and thus the appearance of Sup35 N-terminal fragments of different sizes (Figure 1A and Supporting Information Table S1). The shortest fragment (near 7 kDa) should be produced in the presence of the *sup35-240* mutation which is localized in the first third of the *SUP35* gene. The fragment of 15 kDa is expected in cells with *sup35-74*: it contains the entire N-domain of Sup35 with an additional six amino acid residues. Cells bearing the *sup35-218* mutation should contain 20 kDa fragment combining the N-terminal domain with half of the Sup35 M-domain. Finally, cells with *sup35-21* are expected to contain the longest Sup35 fragment with molecular weight near 50 kDa (Figure 1A). 

In the first stage of the work we obtained [*psi^−^*] strains bearing mutant *sup35-n* alleles. For this purpose, the a8-7A-D832 strain (Table S2) was transformed by pRSU1 plasmids bearing *sup35-n*. Transformants were replica plated to 5-FOA medium to select against the plasmid bearing *SUP35* wild-type allele. Yeast cells containing only one plasmid with *sup35-n* exhibited the ability to suppress *ade1-14*, *his7-1,* and *lys2-739* nonsense mutations (Figure 1B).

We then used SDS-PAGE to estimate the amount of full-length Sup35 and its fragments in yeast haploid cells bearing mutant alleles of *SUP35*. A decreased amount of full-length Sup35 was demonstrated for all mutations (Figure 1C). The lowest amount of Sup35 was detected for *sup35-240* mutant that corresponds to our previous data [2]. This amount of the protein was supposed to be insufficient for cell viability. One possible explanation for this paradox is that amino acid, incorporated by readthrough of UAA stop codon in *sup35-240* mutant, ameliorates properties of Sup35. To address this question experimentally we have analyzed effects of lysine and tyrosine substitutions of 56th glutamine on Sup35 stability and functionality, since it was found that glutamine (Q), tyrosine (Y), and lysine (K) are inserted in UAA codon in yeast [11]. 7A-D832 cells contained plasmids bearing *sup35* mutations, leading to the Q56K or Q56Y substitutions were grown to logarithmic phase and cycloheximide was added up to 100 µg mL^−1^. Sup35 level was estimated after 0, 4, and 20 h of incubation, and Adh1 was used as the internal control. It was shown that Sup35 is a stable protein with an approximate half-life more than 2 h [12] or even near 33 h [13], for this reason we used prolonged (20 h) incubation with cycloheximide. The degradation rate of Sup35-Q56Y protein was equal to that of wild-type Sup35, whereas Sup35-Q56K protein was more stable (Figure S1A). We also tested the effect of mutated Sup35 on cells’ sensitivity to paromomycin, an antibiotic that influences the translation fidelity and phenotypically suppresses nonsense mutations in yeast. One of the pleiotropic effects of *sup35* mutations is a decreased growth on media with this antibiotic (reviewed in [14]). Both wild-type and Sup35-Q56Y strains were able to grow on media with 0.25 mg mL^−1^ of paromomycin. However, Sup35-Q56K was able to tolerate significantly higher concentrations of paromomycin (up to 1 mg mL^−1^) (Figure S1B). Thus full-length Sup35 synthesized in the case of PTC readthrough can function better than the native Sup35. This may account for all other full-length Sup35 proteins synthesized in *sup35-n* mutants. It should be also noted that both the Q56K and Q56Y substitutions did not possess any suppressor phenotype (Figure S1B).

In the case of cells containing *sup35-21, sup35-74,* or *sup35-218* mutations, short N-terminal fragments of Sup35 were detected, and the size of the fragments corresponded to what was expected (50, 15, and 20 kDa, respectively). For the *sup35-240* mutation, the 7 kDa fragment was never detected. This can be explained by the fact that the antibodies used to detect Sup35 do not recognize the epitope located in the N-terminal fragment of the Sup35. Alternatively, Sup35-240 can degrade rapidly, and it was shown that the NM-domain of Sup35 is a substrate for proteasome [12]. Nevertheless, we supposed that Sup35-240 is presented in corresponding cells, because both *sup35-240* mutation or overproduction of Sup35-240 fused to GFP lead to the prion elimination (see below). 

For further investigation of the effects of discussed N-terminal Sup35 fragments on [*PSI^+^*] we needed to estimate their stability after blocking protein synthesis through cycloheximide treatment. 7A-D832 transformants expressing either *SUP35* or *sup35-n* mutant alleles were grown to logarithmic phase and cycloheximide was added up to 100 µg mL^−1^. Sup35 level was estimated after 0, 4, and 20 h of incubation. According our data Sup35-21 and Sup35-218 fragments are stable, they are still detectable after 4 h incubation with cycloheximide (Sup35-21) and even after 20 h (Sup35-218) (Figure 1D), but we could not detect Sup35-74 fragment in this experiment. For this reason and in order to test a stability of Sup35-240, we repeated cycloheximide chase assay using a chimeric construction in which short Sup35 fragments produced from the *sup35-240* and *sup35-74* alleles were fused with GFP. Sup35-240-GFP which we could detect only with anti-GFP antibody has shown the same stability as wild-type Sup35 (Figure 1E, upper panel). Sup35-74-GFP was recognized by both anti-Sup35 and anti-GFP antibodies and was stable after 20 h incubation with cycloheximide (Figure 1E, middle panel). The stability of chimeric Sup35-240-GFP and Sup35-74-GFP proteins could not be explained by a stabilization effect of fused GFP, because GFP alone is less stable than both chimera (Figure 1E, lower panel). Our data are consistent with the previous estimation of an approximate half-life 7 h for wild-type GFP in yeast [15]. It should be also mentioned that the amount of Sup45 in *sup35-n* mutants was similar to the wild-type cells (Figure 1F), that is in agreement with our previous data [2].

To characterize effect of *sup35-n* mutations in diploids, derivatives of a8-7A-D832 [*psi^−^*] strain, containing *sup35-n*, were mated with an isogenic 7A-D832 [*psi^−^*] strain, bearing *SUP35*. Resulting diploids retained an omnipotent nonsense suppressor phenotype (Figure S1C), did not significantly differ by Sup35 amount and were characterized by equal amount of Sup45 (Figure S1D). To test whether these [*psi^−^*] diploids bearing two plasmids contain also short N-terminal Sup35 fragments their lysates were analyzed by Western blotting. No such fragments were detected while in strains with only one mutant plasmid they were visible in case of *sup35-21* or *sup35-218* and poor detected for *sup35-74* (Figure S1E). 

### 2.2. Combination of sup35-n Mutations with the [PSI^+^] Prion in Haploid and Diploid Strains

**Figure 2:**
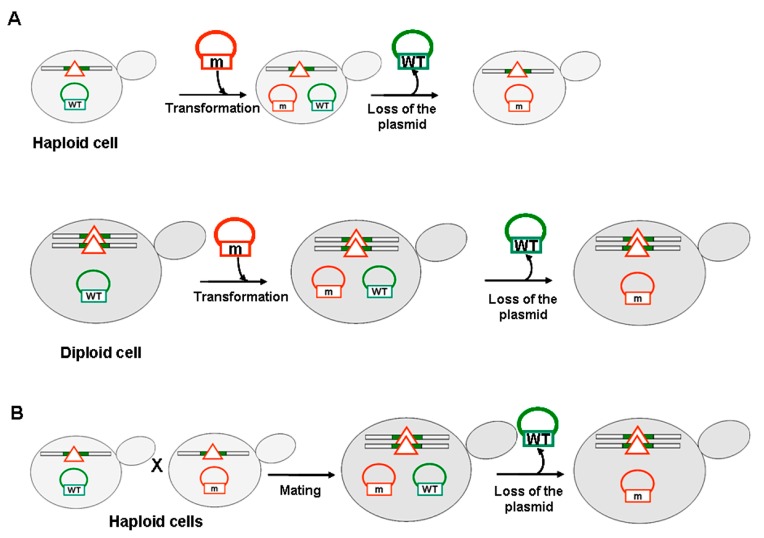
Experimental approaches used to combine *sup35-n* mutations with the [*PSI^+^*] prion. (**A**) Haploid [*PSI^+^*] strain containing wild-type *SUP35* gene on the plasmid, was transformed with *sup35-n* bearing plasmid; resulting transformants were replica plated on 5-FOA medium to select against the [*URA3 SUP35*] plasmid (upper panel). The same procedure was used for diploid [*PSI^+^*] strains (lower panel). (**B**) Haploid [*PSI^+^*] strain, containing wild-type *SUP35* gene on the plasmid, was mated to derivatives of a [*psi^−^*] strain bearing *sup35-n* plasmid; resulting diploids were replica plated to 5-FOA medium to select against the [*URA3 SUP35*] plasmid. In all cases isogenic [*psi^−^*] strains were used as a control. *SUP35* wild-type allele (WT) is marked in green, while the mutant *sup35-n* allele (m) is marked in red. A red triangle indicates non-functional *SUP35* gene on the chromosome.

Previously we showed that nonsense alleles of *SUP45* exhibit synthetic lethality with the [*PSI^+^*] prion [9]. The purpose of the current study was to evaluate the effect of the *sup35-n* mutant alleles on the viability of yeast cells bearing the [*PSI^+^*] prion. We took advantage of a yeast experimental system which allows us to utilize both haploid and diploid strains to identify mechanisms of synthetic lethality which are impossible to detect at a haploid level (see below). Two experimental approaches to combine *sup35-n* mutations with the [*PSI^+^*] prion were used: (i) plasmid shuffle (Figure 2A) and (ii) mating (Figure 2B). In the first approach *SUP35* plasmid was shuffled by *sup35-n* plasmid; these experiments were carried out both on haploid and diploid strains (Figure 2A). In the second approach a haploid [*PSI^+^*] *SUP35* strain was mated to a haploid [*psi^−^*] *sup35-n* strain whereupon the plasmid with *SUP35* was lost (Figure 2B). In all the experiments, a comparison with isogenic [*psi^−^*] strains was made. The [*PSI^+^*] strain 10-7A-D832 used in this work contains a “strong” variant of [*PSI^+^*] as implied by phenotype comparison with well-characterized OT56 or OT55 strains carrying “strong” and “weak” variants of [*PSI^+^*] respectively ([16] and references therein) (Figure S2).

### 2.3. Prion [PSI^+^] Persists in Strains with sup35-n Mutations and Wild-Type Allele of SUP35

In order to investigate the effect of *sup35-n* mutations on their ability to coexist with the [*PSI^+^*] prion, isogenic haploid strains 7A-D832 [*psi^−^*] and 10-7A-D832 [*PSI^+^*] were transformed by plasmids carrying the *sup35-n* mutant alleles. It should be noted that the transformation of the [*PSI^+^*] strain was characterized by less efficiency and transformants had slower growth compared to the [*psi^−^*] strain (Figure S3). Growth inhibition in the case of a wild-type strain (*WT/WT* in Figure S3) can be explained by the prion toxicity induced by an increased amount of Sup35 and sequestration of soluble Sup35 in [*PSI^+^*] aggregates (reviewed in [6]). However, the transformation with plasmids containing *sup35-n* mutations will not significantly increase the overall amount of Sup35. Interestingly, transformants containing *sup35-240* were characterized by increased growth compared to wild-type and other mutations (compare *WT/sup35-240* with other transformants in Figure S3).

**Figure 3:**
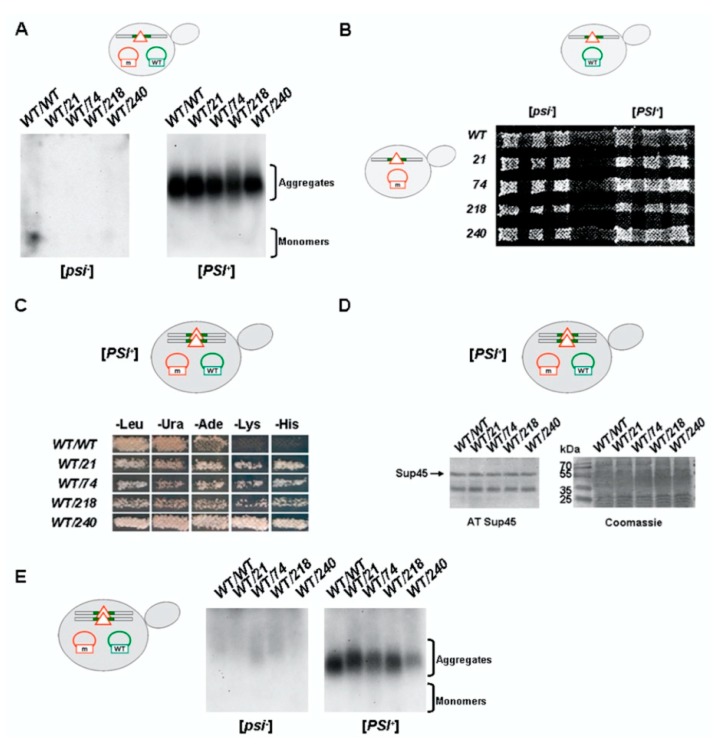
Nonsense mutations in the *SUP35* gene do not affect [*PSI^+^*] maintenance in the presence of wild-type *SUP35*. (**A**) Yeast cell lysates from transformants containing the plasmid with the *SUP35* gene in combination with the plasmid carrying the indicated mutant allele were characterized by SDD-AGE followed by Western blotting with anti-Sup35 antibodies. (**B**) Mating of [*psi^−^*] or [*PSI^+^*] haploid cells bearing plasmid with wild-type *SUP35* (vertical lines) with [*psi^−^*] cells containing mutant plasmids (horizontal lines) leads to the formation of viable [*psi^−^*] and [*PSI^+^*] diploids after 5 days of incubation at 30 °C. (**C**) *SUP35*/*sup35-n* [*PSI^+^*] diploids are able to grow on media selective for nonsense suppression after 5 days of incubation at 30 °C. Media without leucine (-Leu) or uracil (-Ura) were used to control the presence of both plasmids. Ten independent transformants were tested in each case, representative results are shown. (**D**) Diploid [*PSI^+^*] strains containing two plasmids have the equal Sup45 amount. The same transformants as on the Panel C were tested by Western blotting. Coomassie staining of the same gel was used as a loading control. (**E**) Yeast cell lysates from diploids shown on the Panel B were characterized by SDD-AGE with immunoblotting for Sup35.

We demonstrated that *sup35-n* alleles are dominant mutations that lead to the suppression of all nonsense mutations studied (Figure S1C), in particular *ade1-14.* This mutation is often used to determine the presence of the [*PSI*^+^] prion by growth on a selective medium without adenine or colony color (reviewed in [6]). In our case it was impossible because of the identical suppressor phenotype of *sup35-n* mutations and the [*PSI^+^*] prion. Therefore, SDD-AGE was used to determine the presence of aggregated Sup35 in haploid cells containing the plasmid with the *SUP35* gene in combination with the mutant plasmid. The presence of Sup35 aggregates was confirmed in all the [*PSI*^+^] cells and no such aggregates could be detected in the [*psi^−^*] cells (Figure 3A). Thus, in the presence of the *SUP35* gene, *sup35-n* mutations do not affect [*PSI*^+^] maintenance in haploid cells. 

Next, we tested the possibility of the formation of viable diploids by mating *sup35-n* mutants with the [*PSI^+^*] *SUP35* strain. Derivatives of the [*psi^−^*] a8-7A-D832 strain bearing *sup35-n* plasmid were mated with 10-7A-D832 [*PSI^+^*] strain containing the *SUP35* gene on the plasmid. In all cases viable diploids were formed (Figure 3B). Obtained [*PSI^+^*] diploids were able to grow on selective media for nonsense suppression (Figure 3C), they did not differ in the amount of Sup45 (Figure 3D). SDD-AGE was used to determine the [*PSI*^+^] status of the obtained diploids. All diploid cells containing the plasmid with the *SUP35* gene in combination with the mutant plasmid were characterized by the presence of Sup35 aggregates in the case of [*PSI*^+^] cells and the absence of such aggregates in the case of [*psi^−^*] cells (Figure 3E). Therefore, in diploid strains *sup35-n* mutations in the combination with *SUP35* are compatible with the [*PSI*^+^] prion, in contrast to *sup45-n* mutations.

### 2.4. Nonsense Mutations in SUP35 Lead to Either Lethality or Prion Loss in the [PSI^+^] Haploid Strains

**Figure 4:**
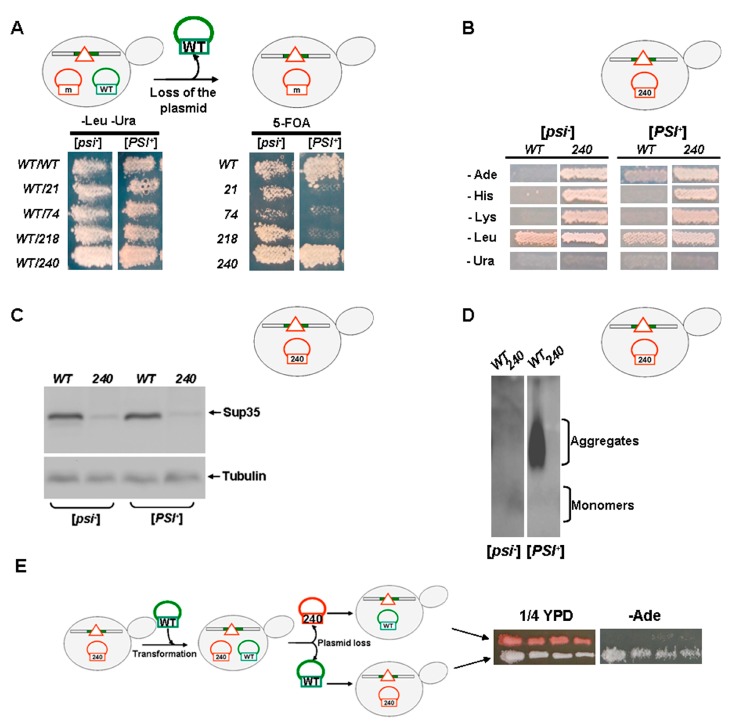
Nonsense mutations in *SUP35* lead to the lethality or the prion loss in the [*PSI^+^*] haploids. (**A**) Combination of the [*PSI^+^*] prion with *sup35-21, sup35-74* or *sup35-218* mutations is lethal. Strains 7A-D832 [*psi^−^*] and 10-7A-D832 [*PSI^+^*] containing two plasmids: one, with wild-type *SUP35*, and the second, with *sup35-n,* were replica plated on 5-FOA media to select against *SUP35* plasmid. Only [*PSI^+^*] cells bearing *sup35*-*240* were able to grow after 5 days of incubation at 30 °C. (**B**) Yeast cells selected as shown on the Panel A, were plated on media lacking adenine (-Ade), histidine (-His), or lysine (-Lys) to test nonsense suppression, as well on media lacking leucine (-Leu) or uracil (-Ura) to confirm the presence [*LEU2*] and absence of [*URA3*] plasmid, respectively. Growth after 5 days of incubation at 30 °C is shown. (**C**,**D**) Yeast cell lysates from transformants shown on the Panel B were characterized by SDS-PAGE (Panel C) or by SDD-AGE (Panel D) with immunoblotting for Sup35. Full-length Sup35 and tubulin are shown by arrows. (**E**) [*PSI^+^*] cells shown on the Panel B were transformed by *SUP35* plasmid, whereupon spontaneous loss of [*sup35-n LEU2*] or [*SUP35 URA3*] plasmids was performed. Growth of cells bearing only one plasmid on 1/4 YPD or adenine-lacking medium after 5 days of incubation at 30 °C is shown.

Haploid transformants containing two plasmids, one with the *SUP35* allele and another with the *sup35-n* mutation, described in the previous section (Figure 3A), were replica plated on a medium containing 5-FOA (see Figure 2A). Only in the case of the *sup35-240* mutation the substitution of the *SUP35* allele in the [*PSI^+^*] strain was observed (Figure 4A). In the isogenic [*psi^−^*] strain, all transformants were able to grow on the 5-FOA containing medium, albeit with different efficiency (Figure 4A).

Cells grown on media with 5-FOA and containing presumably [*sup35-240 LEU2*] plasmid were subcloned and tested for phenotype. Selected cells were able to suppress *ade1-14, his7-1,* and *lys2-739* nonsense mutations both in [*psi^−^*] and [*PSI^+^*] strains (Figure 4B), indicating the presence of *sup35-240* mutation after loss of the plasmid with *SUP35*. This was also confirmed by the SDS-PAGE data (Figure 4C), showing that the [*psi^−^*] and [*PSI^+^*] transformants containing the *sup35-240* plasmid are characterized by a reduced amount of full-length Sup35, unlike the wild-type cells. In order to evaluate the presence of [*PSI^+^*] aggregates in these transformants, SDD-AGE was used. This method showed a complete absence of Sup35 aggregates in the *sup35-240* transformants selected in both [*psi^−^*] and [*PSI^+^*] cells (Figure 4D). Thus, the growth of these transformants in a medium without adenine could not be explained by the presence of the prion, but instead, it is caused by the strong suppressor phenotype of the *sup35-240* mutation. 

To further confirm the presence of the *sup35-240* mutation and the [*PSI*^+^] prion absence, cells bearing [*sup35-240 LEU2*] plasmid were used for re-transformation with [*SUP35 URA3*] plasmid (Figure 4E). After this procedure, one of the plasmids was lost spontaneously. Cells bearing *SUP35* were red on 1/4 YPD medium and Ade^−^, while cells with *sup35-240* were white and Ade^+^ (Figure 4E).

Thus, in [*PSI*^+^] haploid cells plasmids bearing *sup35-21, sup35-74,* and *sup35-218* mutations are unable to substitute plasmid with the *SUP35* gene, leading to lethality of yeast cells in the 5-FOA media. In [*PSI*^+^] cells with *sup35-240* plasmid, only cells that had lost the prion were selected.

### 2.5. Incompatibility of sup35-n with the [PSI^+^] Prion in Diploids Depends on the Technique Used to Obtain Such Diploids

[*PSI*^+^] and [*psi^−^*] diploids containing two plasmids: one with *SUP35* and another with *sup35-n* mutation, were obtained either by transformation of diploid strains (see Figure 2A) or by mating of corresponding haploid strains (see Figure 2B). In both cases, to lose *SUP35* plasmid and to keep only the plasmid with mutant *sup35-n,* the plasmid shuffle on the 5-FOA medium was used.

**Figure 5:**
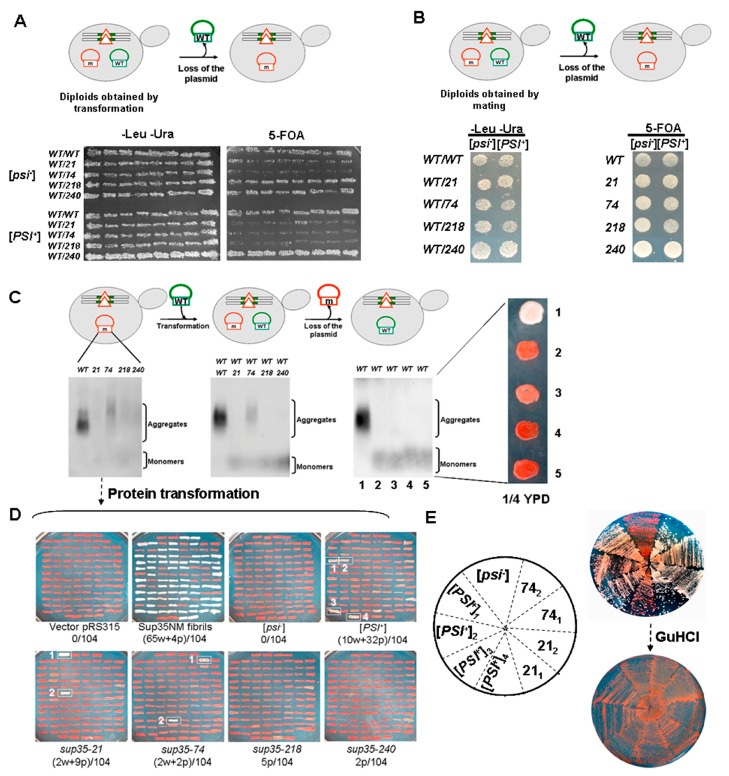
Lethality of the [*PSI^+^*] diploids depends on the technique used to obtain *SUP35*/*sup35-n* cells. (**A**) Cells bearing *sup35-n* mutations are lethal in combination with the [*PSI^+^*] prion in diploid cells obtained by transformation. [*PSI^+^*] or [*psi^−^*] diploids containing plasmid with wild-type *SUP35* were transformed with *sup35-n* mutant plasmids; growth of transformants was tested on the medium selective for both plasmids (left panel), then transformants were replica plated on 5-FOA media to select against *SUP35* plasmid (right panel). Growth of eight independent transformants is shown after 7 days of incubation at 30 °C. (**B**) Cells bearing *sup35-n* mutations are viable in combination with the prion [*PSI^+^*] in diploid cells obtained by the mating of [*PSI^+^*] [*SUP35*] strain with [*psi^−^*] [*sup35-n*] strains. Left panel represents the growth of diploids on the medium selective for both plasmids, right panel shows that all cells are able to lose *SUP35* plasmid on 5-FOA media after 7 days of incubation at 30 °C. Eight independent transformants were tested in each case, representative results are shown. (**C**) The presence of *sup35-n* mutations leads to modification of [*PSI^+^*] aggregates properties in diploid strains. Left, cells selected on the Panel B contain only plasmid with mutation. Middle, these cells were transformed by *SUP35* plasmid. Right, spontaneous loss of [*sup35-n LEU2*] plasmid was performed. Lysates of cells bearing different plasmids were subjected to SDD-AGE followed by immunoblotting for Sup35. The right panel represents the growth of transformants bearing only *SUP35* plasmid after loss of mutant plasmid on 1/4 YPD medium after 5 days of incubation at 30 °C. Numbers correspond to the lanes of the last SDD-AGE gel. (**D**) Infectivity of whole cell lysates of diploid strains D1691 [*psi^−^*], D1692 [*PSI^+^*] bearing *SUP35* or *sup35-n* mutant plasmid (selected as shown on the Panel B), was determined by transformation into 1-OT56 [*psi^−^*] [*PIN*^+^]. Control transformation was performed with either vector pRS316 alone, or with fibrils of Sup35NM. Transformants were selected on SC-Ura media, and then patched on 1/4 YPD. In each case, growth of 104 independent transformants after 5 days of incubation at 30 °C is shown. The number of white (w) or pink (p) clones is shown below. (**E**) White clones indicated by numbers on the Panel D were subcloned on 1/4 YPD followed by growth on GuHCl media and additional subcloning on 1/4 YPD.

In the first case, diploid [*PSI*^+^] or [*psi^−^*] strains bearing a single copy of *SUP35* on the pRSU2 plasmid were transformed by pRSU1 plasmids with *sup35-n* mutations. The phenotype of transformants obtained was characterized and the [*PSI^+^*] prion presence in corresponding strains was proved with SDD-AGE and Western blotting (data not shown). Diploid transformants containing two plasmids were replica plated on a medium containing 5-FOA (see Figure 2A). Substitution of the wild-type *SUP35* gene in the [*PSI^+^*] strain was only detected in the case of the *sup35-240* mutation. In the isogenic [*psi^−^*] strain, all transformants were able to grow on the 5-FOA medium (Figure 5A). It should be mentioned that the efficiency of plasmid shuffle was decreased in [*psi^−^*] cells with *sup35-21* and *sup35-74* mutations, which is similar to the data obtained in haploids (compare Figure 4A and Figure 5A).

In the second case, we used diploids obtained by mating of *sup35-n* mutants with the [*PSI^+^*] *SUP35* strain, described in the previous section (Figure 3C), that contained Sup35 aggregates (Figure 3D). All these strains were able to grow on a 5-FOA medium (Figure 5B), and demonstrated that in these [*PSI^+^*] cells efficient shuffling of *SUP35* on corresponding mutant allele is possible. Cells grown on a medium with 5-FOA and containing presumably [*sup35-n LEU2*] plasmid were subcloned and tested for phenotype. Cells bearing *sup35-n* mutations were able to suppress *ade1-14* and *his7-1* nonsense mutations in [*PSI^+^*] strains (Figure S4), indicating the presence of these mutations after loss of the plasmid with *SUP35*.

SDD-AGE showed a practically complete absence of aggregates in most of the transformants studied (Figure 5C, left). However, some transformants with *sup35-21, sup35-74,* or *sup35-218* plasmids still keep a decreased amount of the prion aggregates together with their increased size (Figure S5A). To prove either the prion loss or its maintenance in diploid cells shown in Figure 5C (left) we repeated a plasmid shuffle assay, but in this case, we replaced a mutant plasmid by the wild-type. Diploid cells, bearing plasmids with *sup35-n*, were transformed by the plasmid with *SUP35*, cells containing two plasmids were selected and then tested by SDD-AGE. A small amount of aggregates with increased size compared to wild-type aggregates was found only in the case of *sup35-74* (Figure 5C, middle). Clones that have spontaneously lost [*sup35-n LEU2*] plasmid were selected and once more tested for the presence of Sup35 aggregates. All of them except one were characterized by the complete loss of aggregates and had a red color on 1/4 YPD (Figure 5C, right). The clone 3 derived from *sup35-74* has a pink color on 1/4 YPD that corresponds to traces of aggregates on SDD-AGE (Figure 5C).

Changes of the Sup35 aggregate size (Figure 5C, left) and the lack of the [*PSI*^+^] suppressor phenotype after presence of *sup35-n* (Figure 5C, right) allowed us to suppose that mutations altered the prion variant. To check this, we transformed [*psi*^−^] cells (the 1-OT56 and 2-OT56 strains were used instead of 7A-D832 because the extremely low transformation efficiency of the last one) with whole cell lysates of strains, presented on Figure 5C (left part). The Sup35NM fibrils obtained in vitro were used as the positive control. As a negative control vector pRS316 alone or lysate of D1691 [*psi^−^*] strain were used. The transformants were selected on the SC-Ura. Their nonsense suppressor phenotype was analyzed on the 1/4 YPD (Figure 5D and Figure S5C). All white and pink clones were curable with GuHCl (Figure 5E and data not shown), indicating that they were [*PSI*^+^]. Also, we found that phenotype of cells transformed with lysates of strains, bearing *sup35-n*, differed from the control (lysate of [*PSI*^+^] strain with *SUP35*) by color (Figure 5D,E and Figure S5B). This observation was in agreement with our initial assumption that investigated mutations alter the prion variants. Surprisingly high heterogeneity of control transformant phenotypes might reveal that the [*PSI*^+^] D1692 strain contains a “cloud of the prion variants” [8]. From this point of view, we can assume that presence of *sup35-n* does not directly alter the [*PSI*^+^] variant, but leads to the selection of specific variants from the existing cloud. Nonetheless this mechanism also may be considered as the change of the prion variant.

Thus, diploid cells obtained by the mating of [*PSI^+^*] strains with [*psi^−^*] strains and containing only plasmid with *sup35-21, sup35-74,* or *sup35-218* mutations are able to maintain the [*PSI^+^*] prion. However, [*PSI^+^*] undergoes changes in its properties, and likely the prion variants, that are manifested by an increase in aggregate size and their loss after changing the mutant allele to the wild-type. Diploid cells containing *sup35-240* mutation lost the [*PSI^+^*] prion.

### 2.6. The sup35-240 Mutation Prevents [PSI^+^] Propagation

**Figure 6:**
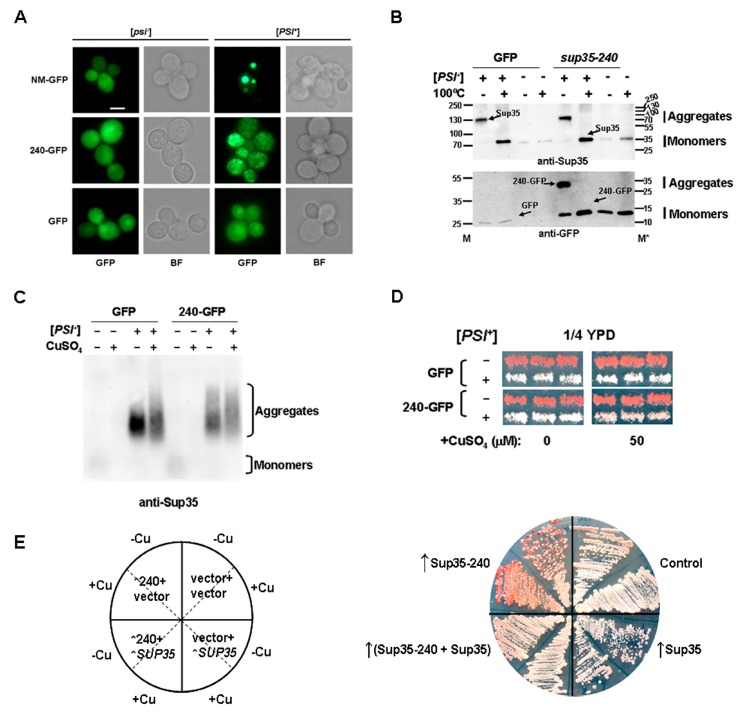
Sup35-240 is included in prion aggregates and leads to destabilization of the [*PSI^+^*] prion. (**A**) 10-7A-D832 [*PSI^+^*] and 7A-D832 [*psi^−^*] strains were transformed with pRS316-pCUP-GFP, pRS316-pCUP-SUP35NM-GFP, or pRS316-pCUP-sup35-240-GFP plasmids. Logarithmic yeast cultures were incubated in the presence of 50 μM CuSO_4_ for two hours. Cells were visualized using fluorescent microscopy. Representative groups of cells are shown. Scale bar corresponds to 5 μm. BF—bright field. (**B**) Lysates of [*PSI^+^*] and [*psi^−^*] yeast cells shown at (A) were subjected to SDS-PAGE with additional boiling followed by immunoblotting with anti-Sup35 (upper panel) or anti-GFP (lower panel) antibodies. M—molecular weight marker (kDa) loaded at the beginning of electrophoresis (M) and after the gel boiling (M*). (**C**) The same [*PSI^+^*] and [*psi^−^*] transformants as in (A) were subjected to SDD-AGE followed by immunoblotting with anti-Sup35 antibodies. (**D**) Transformants shown at (A) were grown on 50 µM CuSO_4_ plates for 3 days, then transferred onto 1/4 YPD media to demonstrate the efficiency of suppression. Six independent transformants were tested in each case. Representative results after 5 days of incubation at 30 °C are shown. (**E**) OT56 ([*PSI^+^*]) strain was transformed with combination of following plasmids: pRS316-pCUP-GFP and pRS315 (vector + vector); pRS316-pCUP-GFP and pRSU1 (vector + *SUP35*); pRS316-pCUP-sup35-240-GFP and pRS315 (240 + vector); pRS316-pCUP-sup35-240-GFP and pRSU1 (240 + *SUP35*). Transformants were grown during 6 days on selective media under non-induced (−Cu) or induced (+Cu) conditions (50 µM CuSO_4_ was used), then transferred onto 1/4 YPD media for 5 days of incubation at 30 °C.

To find out the reason for the prion loss in the presence of *sup35-240* mutation we used a chimeric protein in which short Sup35_1–55_ produced from the *sup35-240* allele was fused with GFP. First, we studied whether such a truncated protein is able to form aggregates in [*PSI^+^*] cells. We used the Sup35NM-GFP fusion protein and GFP as controls. 7A-D832 [*psi^−^*] or 10-7A-D832 [*PSI^+^*] strains were transformed by pRS316 plasmid bearing GFP-fusions under the control of the inducible *CUP1* promoter. We found that overproduced Sup35-240-GFP can decorate pre-existing [*PSI^+^*] aggregates (Figure 6A). However, the [*PSI^+^*] aggregates decorated by Sup35NM-GFP and Sup35-240-GFP were different. Cells containing Sup35-NM-GFP were characterized by the presence of large aggregates, while in cells with Sup35-240-GFP only multiple small foci were detected.

Next, we studied whether Sup35-240-GFP is able not only to co-localize but also to co-aggregate with full-length Sup35 in [*PSI^+^*] strain. For this purpose SDS-PAGE with additional boiling was used [17]. Both in the presence of the control vector with GFP as well as with Sup35-240-GFP [*PSI^+^*] cells containing mostly aggregated Sup35 (Figure 6B, upper panel). Unfortunately, Sup35 antibodies do not recognize the N-terminal fragment of the Sup35, but by using anti-GFP antibodies we have shown that in [*PSI^+^*] cells Sup35-240-GFP is mostly present in the aggregated form (Figure 6B, lower panel). Sup35_1–55_ is possibly included in [*PSI^+^*] aggregates formed by full-length Sup35, followed by a cessation of fibril growth. In this case Sup35-240 can work as an anti-prion agent which will induce the loss of [*PSI^+^*] aggregates. 

SDD-AGE of the same transformants revealed differences in the amount of Sup35 aggregates in the presence of vector-GFP or Sup35-240-GFP (Figure 6C). It seems that short two-hour induction of the *CUP1* promoter resulted in a decrease in the amount of prion aggregates. To confirm the prion loss in the case of *sup35-240* overexpression we compared phenotypic characteristics of cells overproducing Sup35-240-GFP or GFP. 

Transformants of [*PSI^+^*] or [*psi^−^*] cells were grown in the presence of 50 µM CuSO_4_ for three days and then replica plated on 1/4 YPD to check the [*PSI^+^*] status. [*PSI^+^*] cells bearing overexpressed Sup35-240-GFP were characterized by weakened nonsense suppression manifested a dark pink color on 1/4 YPD medium compared to the control [*PSI^+^*] cells (Figure 6D). From this experiment we can conclude that the presence of short Sup35-240 fragment destabilizes the prion. 

However previously we did not observe any changes in [*PSI^+^*] manifestation in diploid cells bearing *SUP35* and *sup35-240*. To analyze this disparity, we transformed [*PSI^+^*] strain (OT56) with the plasmid for Sup35-240-GFP overproduction in combination with either vector pRS315, or centromeric plasmid encoding Sup35 (pRSU1). As negative control combination of empty vectors was used (pRS316-pCUP1-GFP and pRS315) as positive control – pRSU1 plasmid in combination with pRS316-pCUP1-GFP (Figure 6E and Figure S6A). It should be noted that, because of the presence of traces of copper in the medium, the studied effects were almost identical. In this experiment we showed that the additional copy of *SUP35* protect the prion of destabilization caused by transient production of Sup35-240-GFP (Figure 6E and Figure S6A). This observation demonstrated that the effect of *sup35-240* depends on the ratio of full-length and truncated protein and explained the unchanged prion propagation in the diploids. It has been previously shown that overexpression of *SUP35* in [*PSI*^+^] cells leads to the toxic phenotype [18]. Interestingly, this effect is obvious even in the presence of additional copy of the *SUP35* gene on centromeric plasmid. In the presence of Sup35-240 this toxic phenotype completely disappeared (Figure 6E and Figure S6A). When [*PSI*^+^] strain containing vector pRS316 was transformed by multicopy plasmid bearing *SUP35*, transformants were not selected, possibly because of prion toxicity. However, in the presence of pRS316-pCUP1-Sup35-240-GFP few transformants were obtained (Figure S6B). Thus, Sup35-240 fragment destabilize the [*PSI^+^*] prion even in the presence of full-length protein in different strains, but this effect depends of the amount of wild-type Sup35.

## 3. Discussion

### 3.1. [PSI^+^] State and sup35 Mutations

[*PSI*^+^] is a prion that represents an aggregated form of the translation termination factor Sup35 (or eRF3) (reviewed in [6]). The N-terminal domain of Sup35 is responsible for [*PSI*^+^] induction, which is more efficient in the case of artificial introduction in yeast cells of short N-terminal fragments of Sup35 [7,19,20,21]. A generation of N-terminal fragments of yeast Sup35 is possible in vivo in the case of nonsense mutations in the *SUP35* gene. Such mutations lead to the formation of the Sup35 N-terminal fragments (the size of which depends on the position of the premature stop codon (PTC)) together with full-length Sup35 which is synthesized because of readthrough of PTC in the *sup35* nonsense mutant. In the present study we have analyzed the effects of the combination of *sup35-n* mutations with the [*PSI*^+^] prion in haploid and diploid cells. We have found that such effects depend on the presence of wild-type copy of *SUP35,* type of selection as well as the position of nonsense mutation. 

We have demonstrated that unlike *sup45-n*, *sup35* nonsense mutations do not lead to the synthetic lethality of [*PSI*^+^] cells bearing *SUP35* allele. In our experiments, all diploids *SUP35/sup35-n* were viable in the presence of strong [*PSI^+^*] prion and the properties of [*PSI^+^*] were unchanged (at least, those detected by SDD-AGE, Figure 3D). Haploid cells containing two plasmids, one with *SUP35* and another with *sup35-n,* were also viable, retained the [*PSI^+^*] prion and were characterized by unchanged Sup35 aggregates (Figure 3A). Thus, the combination of the wild-type allele of *SUP35* with mutant *sup35-n* does not lead to changes in [*PSI*^+^] properties or its stability. 

Previously we have shown that the main reason for the lethality of *sup45-n* mutants in combination with a strong variant of the [*PSI*^+^] prion is the depletion of Sup45 protein [9]. This is not the case for *sup35-n* mutants that have the same amount of Sup45 as wild-type cells ([2] and this study, Figure 1C).

The new observation in this work is that the synthetic lethality of [*PSI*^+^] combined with *sup35-n* depends on the genetic method used for the creation of such a combination. The transformation of [*PSI*^+^] *SUP35* haploid or diploid cells with *sup35-n* bearing plasmid followed by loss of the *SUP35* plasmid leads to the cell death (except for *sup35-240* that causes the prion elimination and will be discussed below). Thus, we can conclude that in the absence of wild-type copy of *SUP35* nonsense mutations are incompatible with the [*PSI^+^*] prion both in haploid and diploid cells obtained by transformation (Figure 4A and Figure 5A). Nevertheless, the diploid strains with identical combination of *sup35-n* and the prion, obtained by mating, are able to lose the wild-type *SUP35*, retaining only the mutant allele.

The cell death in the combination of *sup35-n* and [*PSI*^+^] is not surprising, considering that the decrease in the amount of essential Sup35 will be unfavorable for yeast cells. Some of the *sup35* nonsense mutants contain a very low amount of the full-length Sup35 compared to the wild-type: 3% for *sup35-21* and 10% for the *sup35-74* (Figure 1C). In [*PSI^+^*] cells most of the Sup35 is present in an aggregated form, while in [*psi^−^*] cells it is soluble [22,23]. According to the estimation by different authors, [*PSI*^+^] cells contain 0.5-2% of soluble Sup35 compared to [*psi^−^*] cells [24,25]. Such an amount of soluble Sup35 is comparable to those in *sup35-n* mutants. However, cells bearing *sup35-n* mutations are not the same as [*PSI*^+^] cells, because they have a low amount of full-length Sup35, together with its short fragments, the role of which is unclear. Furthermore, it was shown that aggregated Sup35 may retain some functional translation termination activity [26]. Thus, one possible reason for the *sup35-n* [*PSI*^+^] cells lethality is the depletion of functional Sup35 protein. Another reason may be connected with an inclusion of short N-terminal Sup35 fragments in aggregates and a subsequent increase in the amount of Sup35 aggregates. Previously it was shown that nonsense mutation *sup35-2* causes lethality in combination with [*ETA^+^*] (a weak variant of [*PSI^+^*]) or [*PSI^+^*] prions [24,27]. Mutation *sup35-2* leads to substitution of TTG for the TAG stop codon, followed by termination of translation and synthesis of 109 amino acid truncated fragments of Sup35 (together with a decreased amount of full-length Sup35) [24]. The lethality could not be overcome by the overexpression of *SUP45* [24,27]. In these experiments lethality was tested in meiotic progeny from crosses of the [*ETA^+^*] strain and the *sup35-2* strain. From these data one can conclude that all [*ETA^+^*] *SUP35/sup35-2* diploids were viable. The lethality of *sup35-2* mutation in combination with [*ETA^+^*] prion was explained by the ease of conformational adaptation by Sup35_1–109_ compared to the full-length Sup35 and subsequent cell death in the condition of deficit of full-length Sup35 [24]. Possibly, Sup35-74, Sup35-218, and Sup35-21 fragments formed from corresponding alleles can be included in the [*PSI*^+^] aggregates that lead to cell death.

All the diploids bearing only *sup35-n* that were obtained by the mating of [*PSI^+^*] *SUP35* cells with [*psi^−^*] *sup35-n* mutants followed by the loss of *SUP35* plasmid were viable. Probably, during the growth of the [*psi^−^*] *sup35-n* cells, additional mutations in other loci were selected. Indeed, previously we have shown that the viability of cells bearing *sup45-n* or *sup35-n* mutations increased after the growth in the absence of the wild-type allele [1,28]. The experiments for identification of such mutations are now in progress. 

### 3.2. Nonsense Mutations in the SUP35 Gene Can Change [PSI^+^] Properties and Variant

All *sup35-n* diploids (except *sup35-240*) retained the [*PSI^+^*] prion after loss of the *SUP35* gene, however, some prion characteristics were changed (Figure 5C,D and Figure S5). At least two molecular mechanisms may be proposed for the explanation of this phenomenon. 

The first, short fragments of Sup35 may be incorporated into prion aggregates with very high efficiency. This, in turn, can lead to the enlargement of Sup35 aggregates. Indeed, it was previously shown that different N-terminal Sup35 fragments (Sup35_1–133_, Sup35_1–154_, and Sup35_1–240_) are able to form [*PSI^+^*]-specific Sup35 aggregates composed solely of the N- or NM-fragments [19]. Nevertheless, from those data it is not clear whether N-terminal Sup35 fragments can be included in the [*PSI^+^*] aggregates formed by the full-length Sup35 or form independent aggregates. From the data of seeding activity of Sup35_1–240_, which was able to convert full-length soluble Sup35 from [*psi^−^*] lysate to its aggregated form [19] one can suggest that this fragment is included in the aggregates. It was shown that truncated N-domains are able to decorate pre-existing Sup35 aggregates in [*PSI^+^*] strains [20]. However, in these experiments Sup35 N-domain fragments were fused with M-GFP, thus their interaction with Sup35 can be different for such of N-domains only. 

The second mechanism assumes that the incorporation of short Sup35 fragments into aggregates affects their fragmentation by cellular chaperones. Thus, the *sup35-n* mutation may lead to an increase in the size of aggregates and a decrease in the propagon number. In turn, daughter cells will receive a lower number of the prion seeds and the [*PSI*^+^] will be weakened. In the case of *sup35-74* (which produces the fragment 1-129 aa) the second mechanism is in good agreement with the identification of the Hsp104 binding site in the Sup35. The Sup35 fragment from 129 to 148 aa is essential for the interaction of these two proteins and [*PSI*^+^] elimination by Hsp104 overproduction. However, Sup35 lacking fragment 129-148 aa is still capable of maintaining the prion [29]. The analogous results obtained for *sup35-218* (fragment 1-180 aa) allow us to suggest that the unknown site for chaperones is located in the missed part of the M-domain (182-256 aa) of the C-domain of Sup35, because the *sup35-21* have no effect on the prion). Additional experiments are needed to discriminate between the above two mechanisms. Therefore, for the first time we have demonstrated that *sup35* nonsense mutations may affect [*PSI*^+^] properties that in turn can lead to the formation of the new prion variant. Protein transformation revealed that the [*PSI*^+^] variants maintained in diploid cells with *sup35-n* differ from the strain with *SUP35* (Figure 5D). The detailed molecular mechanisms of this event need to be investigated.

Interestingly, cells bearing *SUP35*/*sup35*-n plasmids do not contain a detectable amount of truncated Sup35 (Figure S1D). One possible explanation originates from known data that for efficient termination of translation both eRF1 and eRF3 are required [30], and in cells bearing WT/*sup35-n* plasmids together with two genomic copies of *SUP45* the amount of Sup35 is not sufficient to generate anti-suppression. In this case, the truncated Sup35 will not be generated (or its amount will be low and will not be detected in Western blots). However, the situation may be more complicated. Previously in the laboratory of Ian Stansfield it was shown that transcriptional up-regulation of the nonsense allele *sup45-18* leads to increased expression of full-length Sup45, even in the context of the negative feedback loop [31]. Authors could not prove this hypothesis by Western blotting because their antibodies did not recognize the N-terminal part of Sup45, but their data indicate that the presence of the *sup45-n* allele can affect the amount of full-length Sup45. We can assume the existence of a similar mechanism in the case of *sup35-n* mutations.

### 3.3. Possible Influence of Sup35 Interacting Proteins on Viability of sup35-n Mutants in [PSI^+^] Background

Quite a long time ago it was shown that Sup35 interacts not only with Sup45 but also with proteins, participating in translation termination (e.g., PABP, Upf1-3 proteins (components of the nonsense-mediated mRNA decay complex), as well as with some other proteins associated with this process (Mtt1, Itt1, elongation factor EF-2 and Sla1 (reviewed in [14])). During past years, several Sup35 interacting proteins were identified including Dbp5 [32], ABCE1/Rli1 [33], eIF2 and Hcr1 [34], tubulin and Pub1 [35]. The role of some of these proteins in Sup35 prionization was studied. It was shown that *PAB1* overexpression has an anti-suppressor effect on [*PSI*^+^]-mediated suppression and does not influence [*PSI*^+^] stability [36]. During a screen for antiprion factors against [*PSI*^+^] without the overproduction of Upf1 protein was identified, and it was proposed that inhibition of the prion propagation by Upf proteins is explained by their direct interaction with Sup35, either in soluble or aggregated form [37]. The participation of Upf1 together with Pub1 in a [*PSI^+^*] detoxication system was also shown [38].

It is possible that some of the proteins interacting with Sup35 can influence also the viability of strains bearing *sup35* nonsense mutations in the presence of [*PSI^+^*]. For example, anti-suppressor effect of overexpressed *PAB1* in the [*psi^−^*] strain bearing the *sup35-21* mutation (the same mutation that was used in the current work) was demonstrated [36], the same effect was never studied in [*PSI*^+^] background. Previously we have shown that inactivation of either *UPF1* or *UPF2* gene increases viability of *sup35* nonsense mutants in the [*psi^−^*] background [39]. Recent data [37] allow us to propose that this effect is absent in [*PSI*^+^] strain. Nonsense mutation *sup35-21* was employed also in several works studied effects of Dbp5 or Rli1 [32,33] on translation termination, however, [*PSI*^+^] status of the strains remains unknown.

### 3.4. Nonsense Mutation in the SUP35 Gene as a New pnm Mutation

Mutation *sup35-240* is of the greatest interest because its presence in all studied cases led to prion loss. This is the only mutation that could replace the *SUP35* wild-type allele in both haploid and diploid [*PSI*^+^] strains; however, only [*psi^−^*] cells emerged after such a shuffle. The minimal Sup35 fragment that can be incorporated into existing prion aggregates comprises the first 49 residues, its small extension up to the 57th residue increases the efficiency of this process [20]. In our work, using the same approach, we have demonstrated that the short Sup35_1–55_ protein produced from the *sup35-240* allele may be able to decorate the Sup35 aggregates (Figure 6A). Thus, we can suggest that the inclusion of Sup35-240 into prion aggregates can destabilize these aggregates or lead to the formation of non-heritable aggregates. Alternatively, single molecules of Sup35_1–55_ can stick to aggregate ends, and thus interrupt fibril growth. Such a mechanism was proposed to explain the interspecies barrier for prion transmission [40]. We find the first hypothesis more suitable, because the corresponding fragment is incorporated into aggregates with high efficiency (Figure 6A,B). Previously we suggested that in the prion variant studied (yeast strain 10-7A-D832), the first 63-69 residues are included into super-pleated β-structure [41]. This means that Sup35_1-55_ is efficiently incorporated into existing aggregates but cannot template their structure properly, which may lead to the prion destabilization. Unfortunately, we could not detect the untagged Sup35-240 in cells bearing the nonsense mutation (Figure 1C) with available antibodies. Nevertheless, we suppose that the truncated fragment is presented in the cells, because the effects of *sup35-240* and construction *sup35-240-GFP* for the corresponding protein are clearly detected and are the same (Figure 6).

Thus we can conclude that *sup35-240* mutation prevents [*PSI*^+^] propagation and can be considered as a new *pnm* (from “[*PSI*^+^] no more” [42,43]) mutation. The majority of Pnm proteins showed diminished ability to be recruited into the [*PSI*^+^] aggregates in vivo; also, the presence of some mutant proteins led to solubilization of Sup35 aggregates [44]. 

Previously chimeric Sup35_1-61_-GFP protein was used for visualization of pre-existing [*PSI^+^*] prion aggregates [45,46], as well as for prion strains typing [47]. It was shown that prolonged overproduction of the Sup35_1-61_-GFP did not cure [VH-1] or [VK-1], however slight [*PSI^+^*] curing was observed in cells harboring [VL-1] [45,46]. Interestingly, it was also demonstrated that overproduction of Ure2p fragments as well its fusions with GFP cures the [URE3] prion [48,49]. Conversion of the protease-sensitive prion protein (PrP) to its abnormal protease-resistant isoform was inhibited by a peptide containing a conserved PrP sequence both in cell-free system [50] and in tissue culture cells [51].

### 3.5. Possible Applications and Implications

The part of the gene *SUP35* encoding the NM-domains is enriched by potential stop codons (48% compared to 34% for the entire yeast genome) [3]. Therefore, the probability of spontaneous nonsense mutations within the corresponding region is high. Thus, we can propose that *sup35-n* mutations can be found in wild yeast strains. Apparently, combinations of *sup35-n* mutations with [*PSI*^+^] prion are also possible, e.g., by mating of corresponding haploids, or by appearance of spontaneous mutation in diploid strains. However, in most cases [*PSI*^+^] will be quickly eliminated from population. This elimination is explained either by lethality of *sup35-n* combination with [*PSI*^+^] (e.g., *sup35-21*, *sup35-74* and *sup35-218*) or by *pnm*-effect of *sup35-n* itself (e.g., *sup35-240*). In the case if [*PSI*^+^] will be not eliminated by above reasons, it will be weakened and finally disappeared from population.

At least five spontaneous PrP mutations leading to the appearance of a stop codon instead of a sense codon were described (reviewed in [52]). All these mutations lead to synthesis of the C-terminal truncated PrP that lacks the cell surface-linking GPI-anchor. Development of animal models to study the effects of PrP stop mutations has not yet been reported. Possibly, yeast can serve as a model for studying the effects of truncated prion sequences in vivo as well as to propose a potential strategy for the treatment of human and animal prion diseases.

## 4. Materials and Methods

### 4.1. Yeast Strains

Yeast strains used in this study are listed in Supporting Information Table S2. The 7A-D832 [*psi*] strain and its isogenic derivatives, 10-7A-D832 [*PSI^+^*] and a8-7A-D832 [*psi^−^*], with mating type switched to *MATa* were a gift from A. Borchsenius (Department of Genetics and Biotechnology, St. Petersburg State University, St. Petersburg, Russia). Strains contain the *sup35::TRP1* knock-out on the chromosome, compensated by plasmids bearing the *SUP35* gene. OT56 and OT55 isogenic strains [16,53] were used as the [*PSI^+^*]^W^ and [*PSI^+^*]^S^ controls respectively; their isogenic derivate, 1-OT56, was used as [*psi^−^*] control [54]. The diploid strains, D1691 and D1692, *SUP35/sup35-n* or *SUP35/SUP35* were obtained by mating 7A-D832 or 10-7A-D832 with derivatives of a8-7A-D832, bearing plasmids with *sup35-n*.

### 4.2. Plasmids

Plasmids used in this work are listed in Supporting Information Table S3. Positions of *sup35* mutations and resulted substitutions in case of them are shown in Supporting Information Table S1. Plasmid pRS316-pCUP-sup35-240-GFP was constructed by addition of new site for SacII (NEB, Ipswich, MA, USA) into pRS316-pCUP-SUP35NM-GFP after the 166 nucleotide position of *SUP35NM*. The mutations were introduced by site-directed mutagenesis with primers 240_SacII-F and 240_SacII-R (Supporting Information Table S4). This new plasmid was digested by SacII (new site within *SUP35NM* and old one between *SUP35NM* and *GFP*) and self-ligated. Plasmids bearing *sup35* mutations, leading to the Q56K and Q56Y substitutions, were constructed by site-directed mutagenesis. We amplified pRSU1 [55] using highly processive DNA polymerase (AccuPrime Pfx, Invitrogen, Thermo Fisher Scientific, Waltham, MA, USA) with complementary primers, bearing required mutations (Q56Y-f and Q56Y-r or Q56K-f and Q56K-r, Supporting Information Table S4). The following PCR program was used: 95 °C 30 s/55 °C 30 s/68 °C 24 min for 18 cycles. Next, the PCR mixture was treated with DpnI (Thermo Fisher Scientific, Waltham, MA, USA) to remove the template DNA. Then, this solution was used for transformation of *E. coli* DH5α competent cells. All mutations were verified by sequencing in Resource Center “Development of Molecular and Cell Technologies” of St. Petersburg State University, using primers Sup35-BamHI, 112, 113, 116 and sup3 (Supporting Information Table S4).

### 4.3. Growth Conditions and Phenotypic Assays

Standard methods of cultivation and manipulation of yeast and bacteria were used in this work [56,57]. *Escherichia coli* strain DH5α (*supE44 ΔlacU169 (φ80 glacZΔM15) hsdR17 recA1 endA1 gyrA96 thi-1 relA1*) was used for plasmid amplification. Bacterial culture was grown in LB medium with ampicillin (50 µg mL^−1^) at 37 °C. LB medium contained 10 g L^−1^ tryptone, 10 g L^−1^ sodium chloride, 5 g L^−1^ yeast extract (all reagents from VWR Life Science AMRESCO, Radnor, PA, USA). Yeast strains were cultivated at 30 °C in standard solid and liquid media (YPD (rich media), SC (synthetic media), and SC without particular components of SC (selective media). SC media contained 6.7 g L^−1^ yeast nitrogen base w/o a/a (Sigma-Aldrich, St. Louis, MI, USA), 20 g L^−1^ dextrose (VWR Life Science AMRESCO, Radnor, PA, USA), and required amino acids (Sigma-Aldrich, St. Louis, MI, USA). YPD contained 10 g L^−1^ yeast extract, 20 g L^−1^ peptone, 20 g L^−1^ dextrose, and 20 g L^−1^ agar (all reagents from VWR Life Science AMRESCO, Radnor, PA, USA), 1/4 YPD contained 2,5 g L^−1^ yeast extract (or 1/4 of standard YPD medium), 20 g L^−1^ peptone, 40 g L^−1^ dextrose and 20 g L^−1^ agar. 5-FOA media containing 1 mg mL^−1^ 5-fluoroorotic acid (Thermo Fisher Scientific, Waltham, MA, USA) was used for counter-selection of *URA3* plasmids [57]; 1⁄4 YPD medium [58] was used for monitoring the red color phenotype. For the *CUP1* promoter induction CuSO_4_ (Sigma-Aldrich, St. Louis, MI, USA) was added to a final concentration of 50 or 100 µM. Yeast transformation was performed as described [59].

### 4.4. Protein Analysis

Cells for protein extraction were grown in liquid media at 30 °C with shaking at 200 rpm until reaching OD_600_ = 0.6-0.8. Yeast cells for analysis of amyloid aggregates were lysed in non-denaturing conditions with glass beads (Sigma-Aldrich, St. Louis, MI, USA) [17]. The modified alkaline lysis method [60,61] was employed for analysis of proteins amount with SDS-PAGE. Briefly, cells were pelleted and washed twice in water. Cell pellets were incubated for 5 min with 2 M lithium acetate (LiAc, Sigma-Aldrich, St. Louis, MI, USA) in ice. LiAc-treated cells were then centrifuged, the supernatant was aspirated, and the cells were resuspended in 0.4 M NaOH and placed on ice for 5 min. After centrifugation pellets were resuspended in Laemmli buffer (60 mM Tris-Cl pH 6.8, 2% SDS, 10% glycerol, 5% β-mercaptoethanol, 0.01% bromophenol blue) (all reagents are from Sigma-Aldrich, St. Louis, MI, USA), boiled for 5 min, and cleared by centrifugation before separation by PAGE. After electrophoresis proteins were eluted onto PVDF membrane (GE Healthcare Life Sciences, Pittsburgh, PA, USA) by semidry transfer and visualized with Western blot hybridization [56]. GeneGnome hardware and software (Syngene, Bangalore, Karnataka, India) was used for imaging; ECL Select Western Blotting Detection Reagent (GE Healthcare Life Sciences, Pittsburgh, PA, USA) was used for chemiluminescent detection of proteins on PVDF membrane. SDD-PAGE with additional boiling [17] was performed to detect Sup35 in the aggregated and soluble fractions. In this case cell lysates obtained by glass beads were loaded on a 10% SDS-PAGE gel and run until the dye front migrated halfway through the resolving gel. The current was stopped and the gel and glass plates were sealed in plastic sheets prior to boiling upright for 10 minutes in a 100 °C water bath. The gels were removed from the plastic sheets and reinserted into the PAGE apparatus, where voltage was re-applied until the dye front migrated to the bottom of the gel. Molecular weight marker was loaded twice: before gel boiling and after it. Upon boiling aggregated material that is too large to enter the resolving gel breaks down and enters the resolving gel. Following Western blot, two bands are visible per lane: a lower band of soluble material, and a higher band of insoluble material that was delayed in entering the gel. 

Semi-denaturing detergent-agarose gel electrophoresis (SDD-AGE) was used for the analysis of Sup35 amyloid aggregates, followed by capillary transfer onto PVDF membrane [62,63] and Western blot hybridization [56]. The mix (1:1) of rabbit polyclonal anti-Sup35 (SE4290) and anti-Sup35N (SE4291) [2] antibodies was used to detect Sup35. The anti-Sup45 (SE-45-2) [64], anti-α-tubulin (Sigma-Aldrich, St. Louis, MI, USA) and Anti-Tag(CGY)FP (Evrogen, Moscow, Russia) antibodies were used for hybridization with corresponding proteins. ECL Prime Blocking Reagent (GE Healthcare Life Sciences, Pittsburgh, PA, USA) or defatted powdered milk in Tween Tris-buffered saline (TTBS) was used for PVDF membrane blocking (1% w/v and 5% w/v, respectively). The same reagents (0.1% w/v and 5% w/v, respectively) were used as diluent buffer for antibodies. For quantification of Sup45 amount Criterion Stain Free gels (Bio-Rad, Hercules, CA, USA) or Coomassie R250 (Sigma-Aldrich, St. Louis, MI, USA) staining of membranes were used. To test a protein stability cells were grown to logarithmic phase, then the cycloheximide (Sigma-Aldrich, St. Louis, MI, USA) was added into the media to the final concentration 100 µg mL^−1^. Cycloheximide-treated cells were harvested at different time points (0, 4, 6, 8, 10 and 20 hrs) and processed for immunoblotting with anti-Sup35 or anti-GFP antibodies. 

Usually 30-50 µg of total protein per lane was loaded depending on the experiment. To determine the protein concentration in the lysates we performed Bradford assay using Quick Start™ Bradford 1x Dye Reagent (Bio-Rad, Hercules, CA, USA) according to the manufacturer recommendations. The standard protocol for 250 µl microplate assay was used.

### 4.5. Protein Transformation

Protein transformation was performed based on previously published protocol [65,66] with subsequent modifications [67]. To prepare spheroplasts for transformation, yeast cells were grown in YPD media to OD_600_ = 0.3 and successively washed with sterile H_2_O, buffer (1 M sorbitol, 25 mM EDTA, 50 mM dithiothreitol), 1 M sorbitol, and SCE buffer (1 M sorbitol, 1 mM EDTA, 10 mM sodium citrate, pH 5.8) (all reagents are from Sigma-Aldrich, St. Louis, MI, USA). Cells were spheroplasted with 50 µl of zymolyase (120 U mL^−1^) (Sigma-Aldrich, St. Louis, MI, USA) in SCE buffer at 30 °C for 30 min. Spheroplasts were successively washed with 1 M sorbitol and STC buffer (1 M sorbitol, 10 mM CaCl_2_, 10 mM Tris, pH 7.5) (all reagents are from Sigma-Aldrich, St. Louis, MI, USA). Pelleted cells were resuspended in 1 mL of STC buffer. A 100 µl portion of the spheroplast suspension was mixed with 2 µl of salmon sperm DNA (5 mg mL^−1^) (Sigma-Aldrich, St. Louis, MI, USA), 500 ng of selectable plasmid (pRS316) and 5 µl solution containing prion particles, either from cell extracts or in vitro-formed Sup35NM filaments. The mixture was incubated during 30 min at room temperature. Fusion was induced by addition of 900 µl of PEG buffer (20% (w/v) PEG 8000, 10 mM CaCl_2_, 10 mM Tris, pH 7.5) for 30 min (all reagents are from Sigma-Aldrich, St. Louis, MI, USA). Cells were centrifuged, resuspended in 150 µl of SOS buffer (1 M sorbitol, 7 mM CaCl_2_, 0.25% yeast extract (Difco BD, New York, NY, USA), 0.5% bacto-peptone (Difco BD, New York, NY, USA)), incubated at 30 °C for 30 min and plated on synthetic media (SC-Ura, 1 M sorbitol, 2% agar) overlaid with top agar (SC-Ura, 1 M sorbitol, 3% agar (Difco BD, New York, NY, USA)). For preparation of yeast lysates standard glass beads method was used [17]. Concentration of total protein in the yeast extracts was measured by Bradford assay. For transformations involving extracts, the final concentration of total protein of the crude yeast extract was 25 µg mL^−1^. Sup35NM fibrils in concentration 0.5 µg mL^−1^ were provided by E. Maksiutenko (Department of Genetics and Biotechnology, St. Petersburg State University, Russia).

### 4.6. Fluorescence Microscopy 

Cultures for fluorescence microscopy were grown on selective media at 30 °C with shaking at 200 rpm until reaching OD_600_ = 0.2-0.4, then CuSO_4_ was added to final concentration of 50 µM. Thereafter cells were incubated for 2 h and then gently pelleted (400 rcf). Cells were rinsed with water and then resuspended in 25% v/v glycerol. Fluorescent imaging was performed using Zeiss Axioscope A1-wide field fluorescent microscope. Images were obtained using the QIClick-F-CLR-12 (QImaging, Surrey, BC, Canada) camera and QCapture Pro 7 software (QImaging, Surrey, BC, Canada).

### 4.7. Statistical Analyses

All experiments were done at least at three biological replicates. The quantification of Western blotting was performed with ImageJ software [68]. To evaluate the differences, Wilcoxon rank-sum test was performed in R for statistical analysis [69]. Differences were considered statistically significant at *p* < 0.05 levels. 

## Supplementary Materials

Supplementary materials can be found at https://www.mdpi.com/1422-0067/21/5/1648/s1.
