# Supplementary material for: Nonsense Mutations in the Yeast SUP35 Gene Affect the [PSI+] Prion Propagation
Source: Int J Mol Sci. 2020 Feb 28;21(5):1648. doi: 10.3390/ijms21051648 (PMC7084296; doi:10.3390/ijms21051648)
Supplement: Supplementary file 1 [file ijms-21-01648-s001.pdf]

International Journal of Molecular Sciences

MDPI

Nonsense mutations in yeast *SUP35* gene change the properties of the [*PSI*$^+$] prion

Nina P. Trubitsina[1], Olga M. Zemlyanko[1,2], Stanislav A. Bondarev[1,2] and Galina A. Zhouravleva[1,2]*

[1]Dept. of Genetics and Biotechnology and [2]Laboratory of Amyloid Biology,
Saint Petersburg State University, St Petersburg, Russia

*Corresponding author.
Correspondence should be addressed to: Galina A. Zhouravleva, Department of Genetics and Biotechnology, St. Petersburg State University, Universitetskaya nab. 7/9, St. Petersburg 199034, Russia
e-mail
zhouravleva@rambler.ru
g.zhuravleva@spbu.ru
tel +7 9119384080

Supporting Information

Supporting Information tables – pp. 2-3

Supporting Information figures and legends – 4-10

Supporting information tables

Table S1. The characteristic of *SUP35* alleles used in this study

SUP35 allele	Nucleotide position	Mutation	Amino acid position	Codon substitution	Size of the short fragment (aa)
sup35-240	166	C → T	56	Gln (CAA) → (TAA)	55
sup35-74	388	C → T	130	Gln (GAA) → (TAA)	129
sup35-218	541	G → T	181	Glu (GAA) → (TAA)	180
sup35-21	1264	C → T	422	Gln (CAA) → (TAA)	421

Table S2. Yeast strains used in the study

Strain	Genotype	Reference
7A-D832	*MATα ade1-14 his7-1 leu2-3,112 lys2-739 trp1-289 ura3-52 SUP35::TRP1* [pRSU2] [*psi*$^-$] [*PIN*$^+$]	A. Borchsenius (unpublished data)
10-7A-D832	*MATα ade1-14 his7-1 leu2-3,112 lys2-739 trp1-289 ura3-52 SUP35::TRP1* [pRSU2] [*PSI*$^+$] [*PIN*$^+$]	
a8-7A-D832	*MAT***a** *ade1-14 his7-1 leu2-3,112 lys2-739 trp1-289 ura3-52 SUP35::TRP1* [pRSU2] [*psi*$^-$] [*PIN*$^+$]	
OT56	*MAT***a** *ade1-14 trp1-289 his3-200 leu2-3,112 ura3-52* [*PSI*$^+$]^S [*PIN*$^+$]	[1,2]
OT55	*MAT***a** *ade1-14 trp1-289 his3-200 leu2-3,112 ura3-52* [*PSI*$^+$]^W [*PIN*$^+$]	
1-OT56	*MAT***a** *ade1-14 trp1-289 his3-200 leu2-3,112 ura3-52* [*psi*$^-$] [*PIN*$^+$]	[3]
2-OT56	*MAT***a** *ade1-14 trp1-289 his3-200 leu2-3,112 ura3-52* [*psi*$^-$] [*pin*$^-$]	
D1691	*MAT***a**/*α ade1-14/ade1-14 his7-1/ his7-1 leu2-3,112/ leu2-3,112 lys2-739/lys2-739 trp1-289/trp1-289 ura3-52/ura3-52 SUP35::TRP1/SUP35::TRP1* [pRSU1] [pRSU2] [*psi*$^-$] [*PIN*$^+$]	This work, mating: a8-7A-D832 [pRSU1] x 7A-D832 [pRSU2]
D1692	*MAT***a**/*α ade1-14/ade1-14 his7-1/ his7-1 leu2-3,112/ leu2-3,112 lys2-739/lys2-739 trp1-289/trp1-289 ura3-52/ura3-52 SUP35::TRP1/SUP35::TRP1* [pRSU1] [pRSU2] [*PSI*$^+$] [*PIN*$^+$]	This work, mating: a8-7A-D832 [pRSU1] x 10-7A-D832 [pRSU2]

Table S3. Plasmids used in this study

Plasmids	Description	References
pRSU1 (pRS315-*SUP35*)	*CEN, LEU2, pSUP35-SUP35*	[4]
pRSU2 (pRS316-*SUP35*)	*CEN, URA3, pSUP35-SUP35*	
pRSU3 (pRS415-*SUP35*)	*LEU2, pSUP35-SUP35*	
pRSU1-21	*CEN, LEU2, pSUP35-sup35-21*	[5]
pRSU1-74	*CEN, LEU2, pSUP35-sup35-74*	
pRSU1-218	*CEN, LEU2, pSUP35-sup35-218*	
pRSU1-240	*CEN, LEU2, pSUP35-sup35-240*	
pRS315	*CEN, LEU2*	[6]
pRS316	*CEN, URA3*	
pRS316-pCUP-GFP	*CEN, URA3, pCUP1-sGFP*	[7] (original name of the plasmid is pRS316CG)
pRS316-pCUP-SUP35NM-GFP	*CEN, URA3, pCUP1-SUP35NM-sGFP*	[7] (original name of the plasmid is pmCUP-NM-GFP)
pRS316-pCUP-sup35-240-GFP	*CEN, URA3, pCUP1-sup35-240-sGFP*	This work

Table S4. Primers used in this study

Name	Sequence (5’ - 3’)
240_SacII-F	CCAAGGTTATTCTGGGTACCCGCGGGGTGGCTATCAACAGTAC
240_SacII-R	GTACTGTTGATAGCCACCCCGCGGGTACCCAGAATAACCTTGG
CFPstart-R	GTGCCCATTAACATCACCATC
Sup35 BamHI	AAGGATCCATGTCGGATTCAAACCAAGG
112	CTTGCTCACCACACATAGCCATATCAAC
113	GGAGGAGAAACCAGTCCAGACTGAAG
116	CATTGGCATTGTTGGTATTATGTGTTG
sup3	GAGCATTGATGTGACGGT
Q56Y-f*	CCAAAATTACCAAGGTTATTCTGGGTACTACCAAGGTGGCTATCAACAGTACAATCCCG
Q56Y-r	CGGGATTGTACTGTTGATAGCCACCTTGGTAGTACCCAGAATAACCTTGGTAATTTTGG
Q56K-f	CCAAAATTACCAAGGTTATTCTGGGTACAAGCAAGGTGGCTATCAACAGTACAATCCCG
Q56K-r	CGGGATTGTACTGTTGATAGCCACCTTGCTTGTACCCAGAATAACCTTGGTAATTTTGG

* nucleotide substitutions are underlined

Supporting Information figures

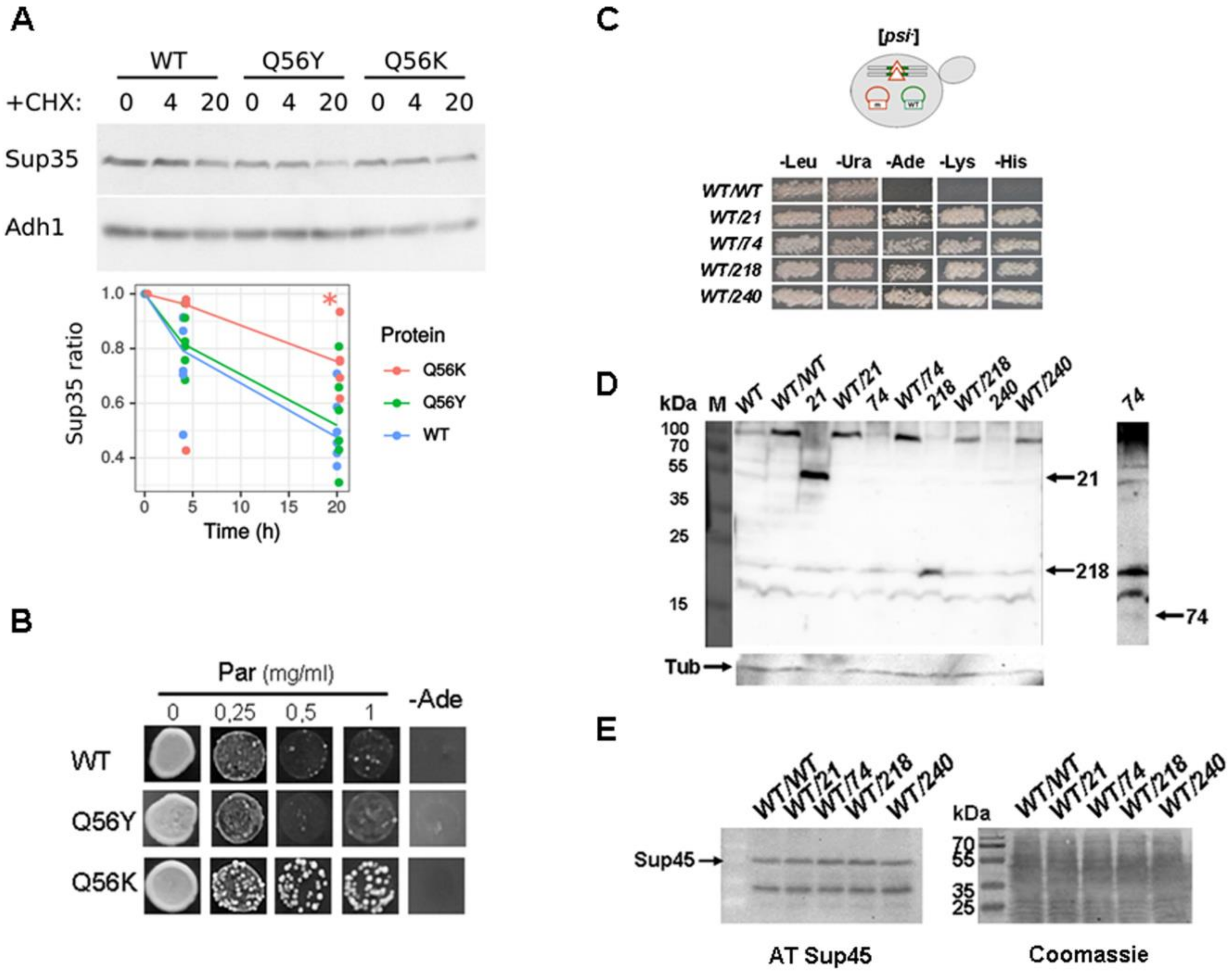

Figure S1. (**A**) Steady-state level of Sup35 in yeast cells containing wild-type copy of Sup35 or missense mutations. Sup35 was estimated in 7A-D832 strain bearing pRSU1 plasmid with either wild-type *SUP35* (*WT*) or one of the mutant *sup35-240Tyr* (Q56Y) or *sup35-240Lys* (Q56K) alleles after incubation in YPD medium containing 100 µg mL^{-1} cycloheximide (CHX) during 0, 4 or 20 hours. The amount of Sup35 in each probe at point 0 was taken as 1.0 and the relative Sup35 amount in other points was calculated. (**B**) Growth of the same transformants as at (A) was assessed on the YPD medium containing different concentrations of paromomycin (Par) after 5 days of incubation at 30 °C. Drops of yeast suspension of the same density were used. Three independent transformants with each plasmid were tested, and representative results are shown. (**C**) The dominant character of *sup35-n* mutations in [*psi*$^-$] diploids containing two plasmids, one with a wild-type *SUP35* and the other with *sup35-n* mutation. Four independent transformants were tested in each case, representative results are shown after 4 days of incubation at 30 °C. Symbols on the left side show combinations of wild-type (*WT*) with *sup35-n* mutations. (**D**) Lysates of the [*psi*$^-$] diploid strains containing one or two plasmids (as indicated by the numbers above the lanes) were analyzed by Western blotting with anti-Sup35 antibodies. M - protein molecular weight marker. Fragments corresponding to the truncated Sup35 are marked by the arrows. Tubulin (Tub) was used as a loading control. An individual lane for *sup35-74* using different contrast to increase intensity of the band corresponding to the short Sup35-74 fragment is shown (taken from the same gel shown at the left). (**E**) Diploid [*psi*$^-$] transformants (the same as at (C)) were tested by Western blotting. Coomassie staining of the same membrane was used as a loading control.

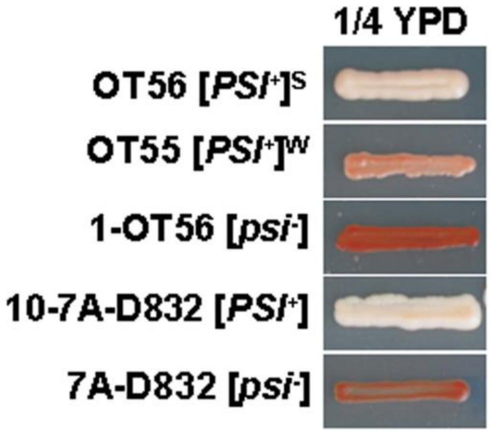

Figure S2. Strain 10-7A-D832 contains a “strong” variant of [*PSI*$^+$]. Growth of isogenic strains OT56 or OT55 carrying “strong” ([*PSI*$^+$]^S) and “weak” ([*PSI*$^+$]^W) variants respectively and strains used in this work on 1/4 YPD medium after 4 days of incubation at 30 ºC.

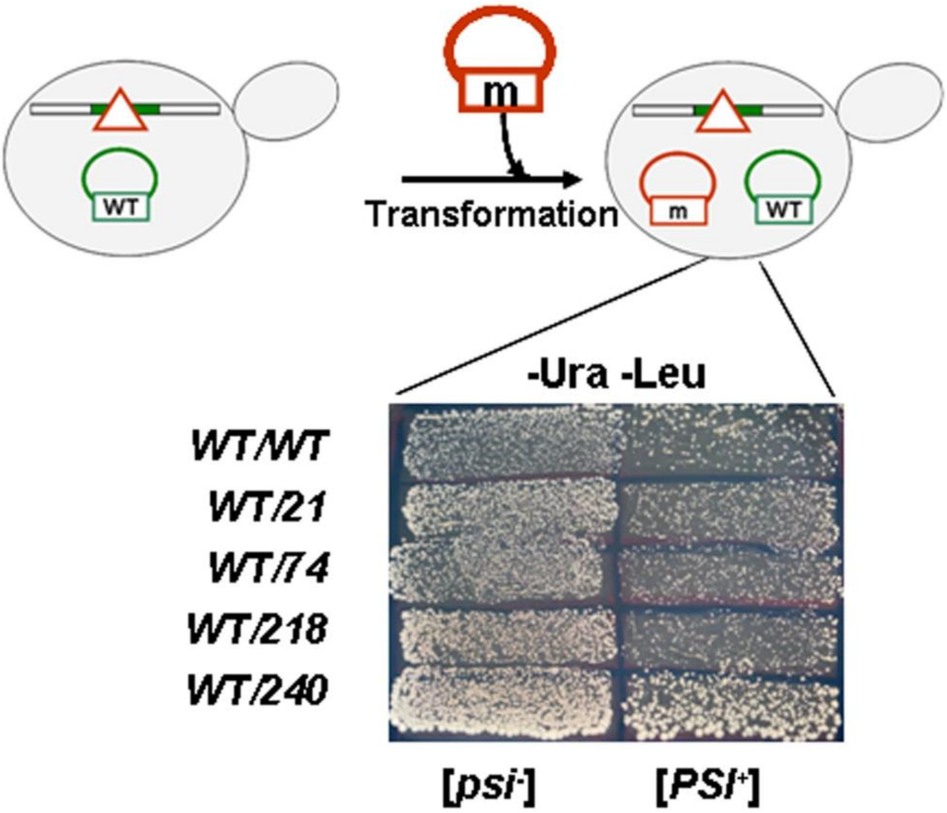

Figure S3. Some *sup35-n* mutations inhibit the growth of corresponding transformants of [*PSI*$^+$] strain after transformation. Strains 7A-D832 [*psi*$^-$] and 10-7A-D832 [*PSI*$^+$] bearing plasmid with wild-type *SUP35* were transformed with *sup35-n* mutant plasmids. Equal amounts of DNA and cells were taken for transformation in each case. After a standard transformation procedure, an equal amount of cells after transformation was plated on media selective for both plasmids. The growth of transformants is shown after 7 days of incubation at 25 ºC.

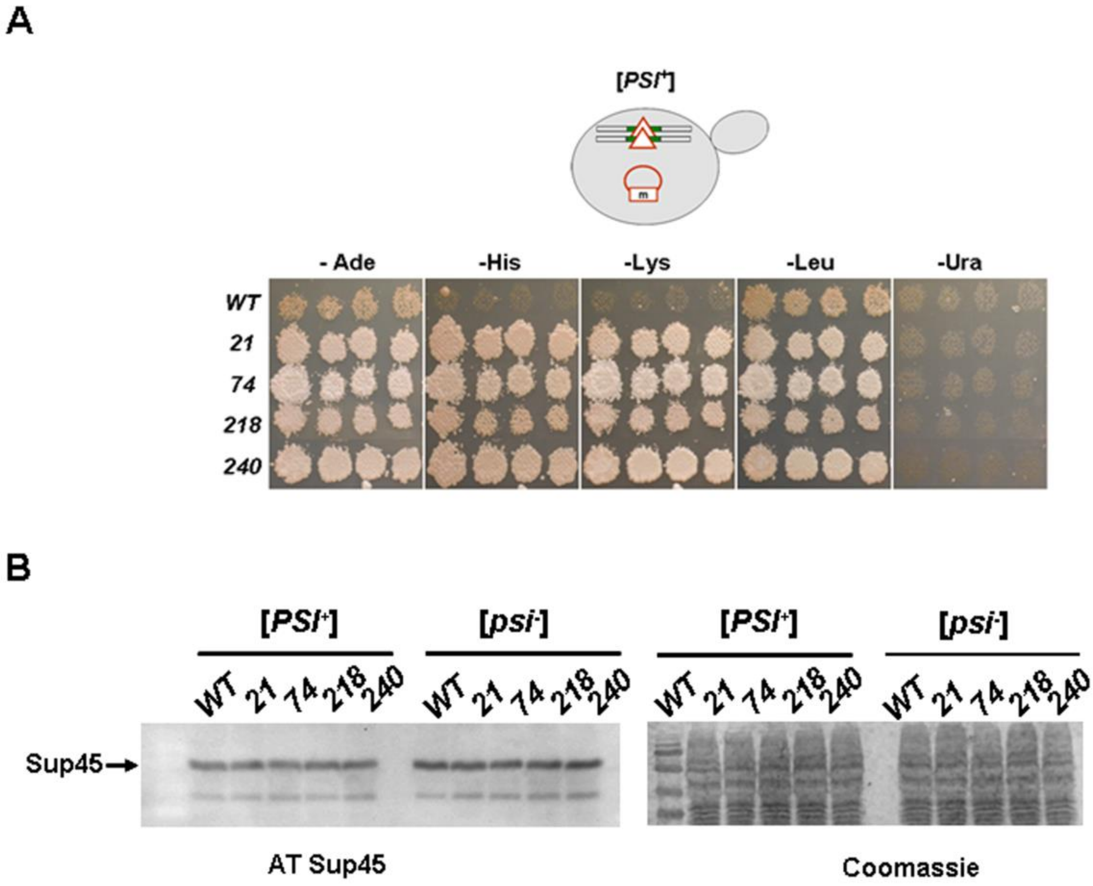

Figure S4. (**A**) Diploid cells bearing *sup35-n* mutations after loss of *SUP35* plasmid are able to grow on selective media for suppression. Yeast cells selected in [*PSI*$^+$] background, as shown in Figure 5B, were plated on selective media to test nonsense suppression, as well on media lacking leucine (-Leu) or uracil (-Ura) to confirm the presence of [*sup35-n LEU2*] and the absence of [*SUP35 URA3*] plasmid, respectively. Four independent clones in each case are shown after 5 days of incubation at 30 °C. (**B**) Diploid [*PSI*$^+$] and [*psi*$^-$] strains containing one plasmid have the same Sup45 amount. The same transformants as at (A) were tested by Western blotting. Coomassie staining of the same membrane was used as a loading control.

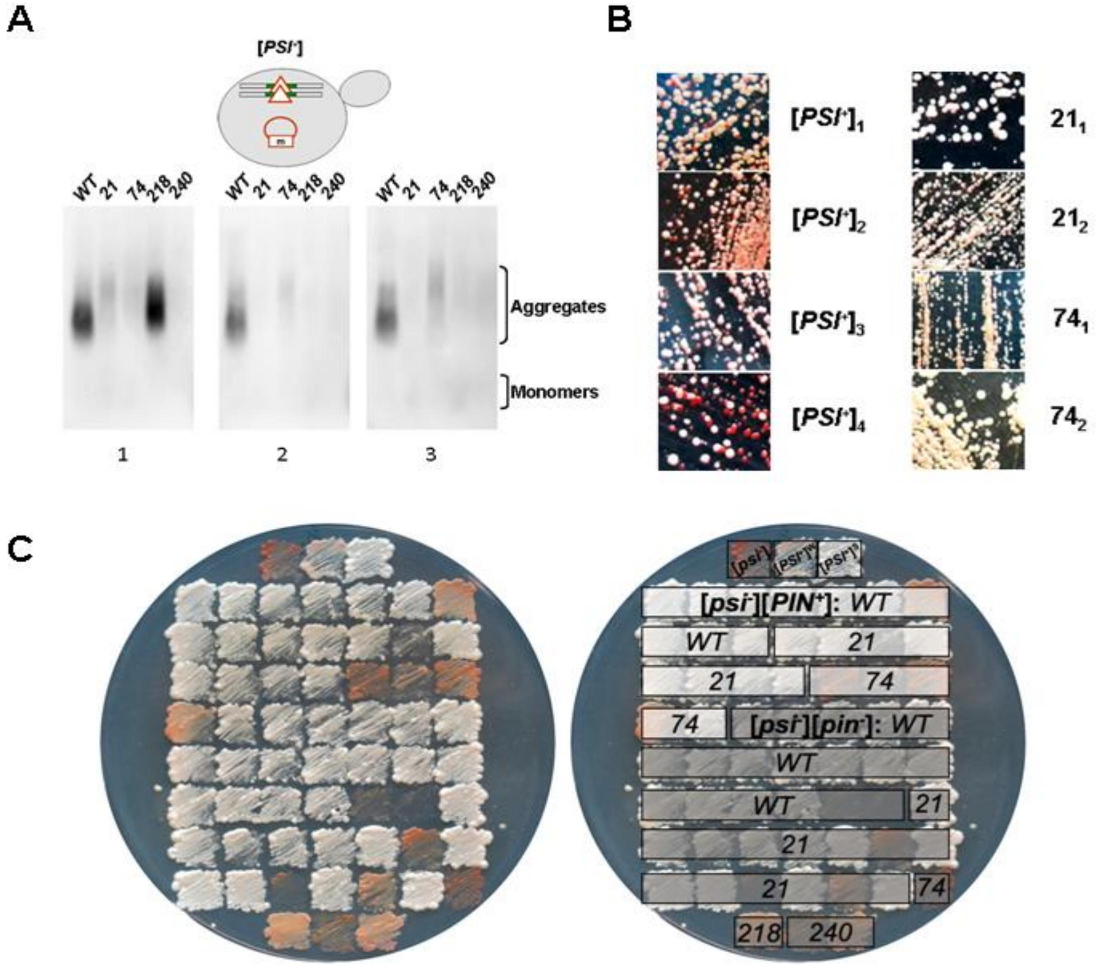

Figure S5. (**A**) [*PSI*$^+$] aggregates change their properties in the presence of *sup35-n* mutations in diploid strains. Cells selected as shown in Figure 5B were used for preparation of lysates followed by SDD-AGE. Three independent sets (1-3) of clones were analyzed. (**B**) The phenotype of subclones of strains presented in Figure 5E. Cells were grown on 1/4 YPD. (C) The phenotype of cells selected after the protein transformation on 1/4 YPD. Infectivity of whole cell lysates of diploid strains D1692 [*PSI*$^+$] bearing *SUP35* or *sup35-n* mutant plasmid (selected as shown in the Figure 5B) was determined by transformation into 1-OT56 [*psi*$^-$] [*PIN*$^+$] (white squares in the left panel), or 2-OT56 [*psi*$^-$] [*pin*$^-$] (grey squares in the left panel). On the top of the panel non-transformed [*psi*$^-$] (1-OT56), weak (OT55) and strong (OT56) [*PSI*$^+$] strains are shown.

I

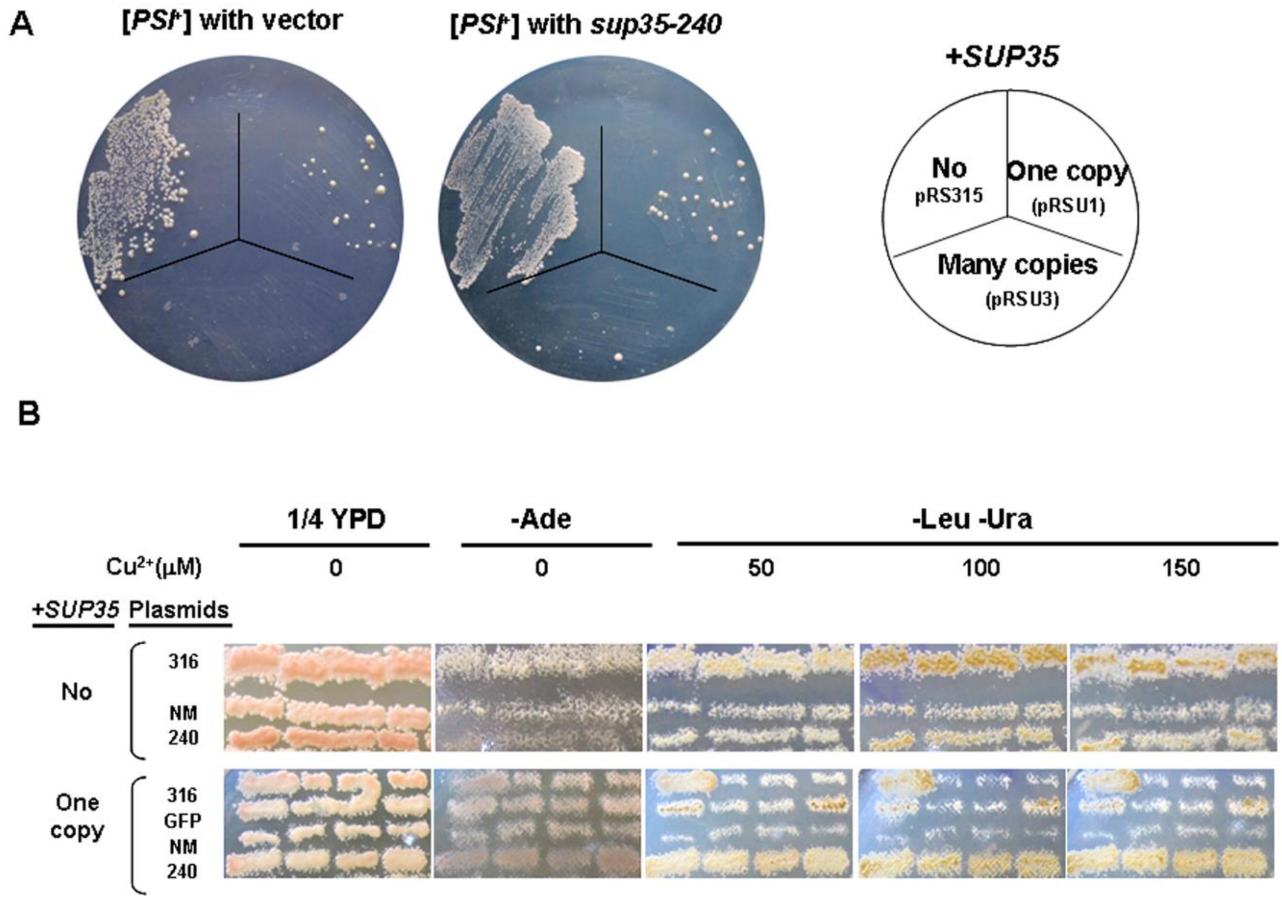

Figure S6. Sup35-240p rescues toxicity associated with the [*PSI*$^+$] prion. (**A**) OT56 ([*PSI*$^+$]) strain transformed with vector pRS316 or with pRS316-pCUP-sup35-240-GFP was re-transformed with pRS315 (control), pRSU1 (one copy of *SUP35*) or pRSU3 (multicopy plasmid containing *SUP35*). Transformants were selected on SC- Leu - Ura. In the case of combination of pRS316 with pRSU3 no transformants were selected, however in the presence of Sup35-240 ten transformants were recovered. (**B**) OT56 ([*PSI*$^+$]) strain was transformed with following plasmids: pRS315 (-*SUP35*); pRSU1 (↑ *SUP35*) or pRSU3 (↑↑ *SUP35*). These plasmids were combined either with vector pRS316 (316), or pRS316-pCUP-GFP (GFP), or pRS316-pCUP-NM-GFP (NM), or pRS316-pCUP-sup35-240-GFP (240). Transformants were grown during 6 days at 30 °C. At the presence of an additional copy of *SUP35* effect of prion toxicity can be seen in all cases except one where pCUP-sup35-240-GFP plasmid was present.

References

1. Derkatch, I.L.; Bradley, M.E.; Zhou, P.; Chernoff, Y.O.; Liebman, S.W. Genetic and environmental factors affecting the *de novo* appearance of the [*PSI*$^{+}$] prion in *Saccharomyces cerevisiae*. *Genetics* **1997**, *147*, 507–519.
2. Newnam, G.P.; Wegrzyn, R.D.; Lindquist, S.L.; Chernoff, Y.O. Antagonistic interactions between yeast chaperones Hsp104 and Hsp70 in prion curing. *Mol. Cell. Biol.* **1999**, *19*, 1325–1333.
3. Matveenko, A.G.; Drozdova, P.B.; Belousov, M.V.; Moskalenko, S.E.; Bondarev, S.A.; Barbitoff, Y.A.; Nizhnikov, A.A.; Zhouravleva, G.A. SFP1-mediated prion-dependent lethality is caused by increased Sup35 aggregation and alleviated by Sis1. *Genes to Cells* **2016**, *21*, 1290–1308.
4. Volkov, K.V.; Aksenova, A.Y.; Soom, M.J.; Osipov, K.V.; Svitin, A.V.; Kurischko, C.; Shkundina, I.S.; Ter-Avanesyan, M.D.; Inge-Vechtomov, S.G.; Mironova, L.N. Novel non-Mendelian determinant involved in the control of translation accuracy in *Saccharomyces cerevisiae*. *Genetics* **2002**, *160*, 25–36.
5. Chabelskaya, S.; Kiktev, D.; Inge-Vechtomov, S.; Philippe, M.; Zhouravleva, G. Nonsense mutations in the essential gene *SUP35* of *Saccharomyces cerevisiae* are non-lethal. *Mol. Genet. Genomics* **2004**, *272*, 297–307.
6. Sikorski, R.S.; Hieter, P. A system of shuttle vectors and yeast host strains designed for efficient manipulation of DNA in *Saccharomyces cerevisiae*. *Genetics* **1989**, *122*, 19–27.
7. Serio, T.R.; Cashikar, A.G.; Moslehi, J.J.; Kowal, A.S.; Lindquist, S.L. Yeast prion [Ψ+] and its determinant, Sup35p. *Methods Enzymol.* **1999**, *309*, 649–673.
